# The refined biomimetic NeuroDigm GEL™ model of neuropathic pain in a mature rat

**DOI:** 10.12688/f1000research.9544.2

**Published:** 2017-05-04

**Authors:** Mary R. Hannaman, Douglas A. Fitts, Rose M. Doss, David E. Weinstein, Joseph L. Bryant

**Affiliations:** 1 NeuroDigm Corporation, Colorado Springs, CO, 80906, USA; 2 Department of Integrative Physiology, University of North Texas Health Science Center, Fort Worth, TX, 76107, USA; 3 Office of Animal Welfare, University of Washington, Seattle, WA, 98195, USA; 4 Department of Biology, University of Colorado, Colorado Springs, CO, 80918, USA; 5 RiverTown Therapeutics Inc., Dobbs Ferry, NY, 10522, USA; 6 Animal Model Division, Institute of Human Virology, University of Maryland School of Medicine, Baltimore, MD, 21201, USA

**Keywords:** animal models, neuropathic pain, erythropoietin, nerve regeneration, neuritis, tissue repair, tissue remodeling, hydrogel, nerve block, morphine resistance, refinement

## Abstract

**Background:** Many humans suffering with chronic neuropathic pain have no objective evidence of an etiological lesion or disease. Frequently their persistent pain occurs after the healing of a soft tissue injury. Based on clinical observations over time, our hypothesis was that after an injury in mammals the process of tissue repair could cause chronic neural pain. Our objectives were to create the delayed onset of neuropathic pain in rats with minimal nerve trauma using a physiologic hydrogel, and characterize the rats’ responses to known analgesics and a targeted biologic.

**Methods:** In mature male Sprague Dawley rats (age 9.5 months) a percutaneous implant of tissue-derived hydrogel was placed in the musculofascial tunnel of the distal tibial nerve. Subcutaneous morphine (3 mg/kg), celecoxib (10 mg/kg), gabapentin (25 mg/kg) and duloxetine (10 mg/kg) were each screened in the model three times each over 5 months after pain behaviors developed. Sham and control groups were used in all screenings. A pilot study followed in which recombinant human erythropoietin (200 units) was injected by the GEL™ neural procedure site.

**Results:** The GEL group gradually developed mechanical hypersensitivity lasting months. Morphine, initially effective, had less analgesia over time. Celecoxib produced no analgesia, while gabapentin and duloxetine at low doses demonstrated profound analgesia at all times tested. The injected erythropoietin markedly decreased bilateral pain behavior that had been present for over 4 months, *p* ≤ 0.001. Histology of the GEL group tibial nerve revealed a site of focal neural remodeling, with neural regeneration, as found in nerve biopsies of patients with neuropathic pain.

**Conclusion:** The refined NeuroDigm GEL™ model induces a neural response resulting in robust neuropathic pain behavior. The analgesic responses in this model reflect known responses of humans with neuropathic pain. The targeted recombinant human erythropoietin at the ectopic neural lesion appears to alleviate the persistent pain behavior in the GEL™ model rodents.

## Introduction and Background

The development of chronic neural pain following tissue injuries in humans is an infrequent but disabling complication ^1– 5^. The persistent pain usually begins gradually, continuing for months to years. Causes of soft tissue injuries include trauma ^6, 7^, such as industrial injuries, surgery, fractures ^8^, sprains, radiation, vibration, and repetitive motion. Disuse after injury or surgery also contributes to the tissue matrix stiffness, edema and pain ^9^. Despite a history of trauma, a trauma-specific neural lesion is seldom found in patients with neuropathic pain symptoms. This absence of an identifiable lesion suggests the involvement of an occult lesion in the initiation and the maintenance of neuropathic pain ^10^, and many of these patients have been theorized to have a peripheral neural “generator” or an “ectopic” site of localized neuroinflammation ^11– 18^.

Occult neural lesions are not usually detectable, as these patients usually do not have clinical evidence of either neural injury or physical abnormalities ^10, 19^. Yet the persistence of pain behaviors in these individuals argues in support of a local neural activation site. *In vivo* peripheral nerve imaging techniques ^20– 25^ and diagnostics are presently being developed ^26, 27^; however, they cannot yet detect abnormalities in small branches of the distal peripheral nerves ^10^, which are the fibers most likely to be affected in soft tissue injuries.

A logical cause for the gradual appearance of chronic pain following soft tissue trauma is the predictable changes that occur during the tissue repair process at the affected site. These changes involve the removal of debris, fibrosis, and the regeneration of damaged tissue, including muscle, nerve, vasculature and extracellular matrix. The gradual remodeling of tissue may result in nerve compression, with delayed onset of pain. One such example of the ability of minimal pressure on the nerve causing severe pain, is trigeminal neuralgia, where even micro-compression of the nerve root can cause severe pain ^28^. The timing of the onset of chronic neuropathic pain parallels tissue morphologic events that occur during healing and tissue remodeling of the affected area ^29^ ( Supplementary file S1: Tissue repair comparison chart). We hypothesize that it is during tissue remodeling that an accumulation of fibrotic matrix (scarring) and possibly local edema alter the neural microenvironment and contribute to the compression of vulnerable nerve cells, resulting in focal neural injury. These injuries can cause atypical matrix forces ^30^ resulting in abnormal function of peripheral glia and neurons. To test this biophysical hypothesis we have created a model of a discrete focal lesion in the rat rear limb that recreates clinical findings found in humans.

Presently, rodent models with neuropathic pain behaviors are created using forms of direct surgical nerve trauma or open surgery with neural irritation using chemicals, drugs, cold or heat ^31, 32^. The most common of these models ^33– 35^ use ligations, neurectomies or a combination to create pain with sensory and motor debility. While these open surgical models are useful in mimicking direct nerve trauma, they do not reproduce the delayed onset of pain behaviors ^36^ without physical deformities as seen in many patients with neuropathic pain after tissue injuries.

Doubts have been raised about whether or not rodents can represent the human condition in neuropathic pain because few effective analgesics have been discovered using them ^1, 37^. We consider the social behaviors ^38^, tissue healing ^39, 40^, and the similarly evoked neural pain behaviors that humans share with rodents as confirmative to the relevance for their use in our study ^41^. We increased the relevance of the rodents by using those of a mature age with a biomimetic lesion ^1, 42^.

Our rodent model is based on clinical observations of patients treated for persistent pain that developed gradually after soft tissue injuries. After a localizing anatomical examination for neuroinflammation, these patients were treated with targeted nerve blocks for regional neuropathic pain (MRH physician practice 1987–2017). The therapeutic blocks used low doses of the neuroprotective agents methylprednisolone or recombinant human erythropoietin to relieve and often resolve neural pain. Our objective in developing the NeuroDigm GEL™ rodent model was to create the delayed onset of neuropathic pain with minimal nerve trauma and characterize the model’s responses to known analgesics and a targeted neuroprotective agent.

## Materials and Methods

### Ethical statement

The protocol was approved by the Institutional Animal Care and Use Committee of NeuroDigm Corporation (IACUC permit number 1-2014/15) and was in compliance with the guidelines of the 8 ^th^ edition of Guide for the Care and Use of Laboratory Animals. All efforts were made to minimize the number of animals used, and pain and suffering. NC3Rs ARRIVE guidelines for reporting on animal research were followed ( Supplementary file S2). 

### Experimental animals

Thirty-seven Sprague Dawley 9.5-month-old male rats (from Harlan facility in Houston, Texas) were received, after being raised within their normal social groups. Their initial weights ranged from 440 to 660 grams, with a mean of 545 grams. In this study, the rats’ human equivalent age is as a mature adult ^43, 44^. The rats had no prior drug exposure. A total of 37 rats were received with 36 rats (minus 1 for neurological injury) enrolled after baseline testing, with GEL *n* = 15, control *n* = 11, sham *n* = 10. Three rats were removed from study for health complications, with 33 finishing study and final group sizes of GEL *n* = 14, control *n* = 11, sham *n* = 8.

### Housing and husbandry

Ventilation and housing were in compliance with the guidelines of the 8 ^th^ edition of Guide for the Care and Use of Laboratory Animals. To limit airborne volatile organic compounds ^45^ a ceiling mounted fresh air exchanger, an air purifier/fan and three activated charcoal reservoirs, changed weekly, were used in addition to standard heating, ventilation and air conditioning. Each rat was housed singly in clear, open cages in the same room. The room and individual cages had ammonia sensors (Pacific Sentry). No other animals or rodents were housed in the facility. The cages were replaced every 2 weeks or earlier. Bedding used were 0.25-inch corncob pellets. Food was LabDiet 5V5R with low phytoestrogens, continuous access. Light-dark cycle was 12 hours, with lights off from 7 PM to 7 AM, except when screening. Maximum lumens at cage level were 20–40; at time of pain behavior testing the maximum lumens were 85–100. The room had no high frequency interference detected (Batseeker Ultrasonic Detector), other than that related to the rats on weekly and as needed testing. Water used was municipal water. In each cage, enrichments were 1) a non-plasticized polyvinyl chloride tube 4” in diameter by 6” in length (Bisphenol A free) for shelter and 2) bedding at an increased depth of 0.75 to 1 inch when dry, to encourage burrowing. Facility was in north Texas. All pain behavior testing was performed in the same room as the rats were housed.

### Study design

**Figure 1.**
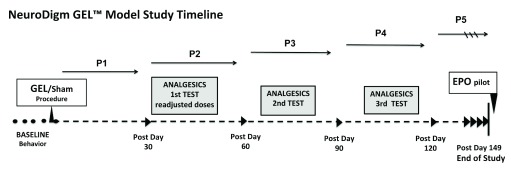
Timeline: Three groups had four drugs screened for analgesia three times over the 5-month study. At the end, a pilot study with localized EPO was performed. Mechanical hypersensitivity testing was performed during and between analgesic testing periods. Each group had the same tests performed. Each group has the same number (n) for all behavioral, analgesic and statistics tests performed, unless stated otherwise. GEL n = 14, sham n = 8, control n = ll.

The eligibility criteria for the rats to be included were: strain Sprague Dawley, male, 9–10 months old, from same housing group at sending facility, no neurological deficits, disease, or prior drug exposure. After receiving, the rats were acclimated for 15 days with subsequent baseline testing ( Figure 1). The rats were housed singly to limit fighting and rough play. The rats were assigned by simple randomization by blindly picking numbered lima beans to one of three groups: GEL procedure, sham procedure, or control (no procedure) with the constraint that the groups would have initially n = 15, 10, 11, respectively. The investigator performing the procedures and behavioral testing was blinded to the rat group assignment and tail identification was masked prior to performance of any procedure. For allocation concealment, another experimenter did the simple random group assignment prior to the initial procedures. The rats were housed in a separate room during the GEL and sham procedures and handed to the investigator by an assistant. The locations of the animals on the rack were randomly changed every 10–14 days. The investigator did not know the group assignments until the unblinding on post procedure day 149.

Isoflurane 2–3% was used for anesthetic induction and during the procedure for approximately 2 minutes. Isoflurane gas was used due to brief anesthetic time needed, enabling less recovery time compared to injectable anesthetics. All animals in each group were screened with four analgesics: morphine, celecoxib, gabapentin and duloxetine, three times each during the 5-month study. The screening involved testing the mid-plantar (tibial nerve) hindpaws of the rats with stimuli to detect mechanical hypersensitivity. Stimuli used were von Frey (light touch) for mechanical allodynia, brush for dynamic mechanical allodynia and pinprick for mechanical hyperalgesia. For all behavioral testing, each rat was placed singly on a wire mesh with manual application of stimuli. The behavioral testing was performed between 1 PM and 11 PM. Animal welfare observations of behavior, coat and movement were checked daily. Monitoring for signs of infection, water and food use were conducted at least three times a week. Weights were documented every 4 weeks, or every 2 weeks as indicated. The same female investigator performed all procedures and screenings, with no one else in the room. On each routine pain behavior test day, after the first six random rats were screened, their results were compared to the prior session of each respective rat, to observe for environmental influences. Subcutaneous injections rather than oral gavage were used for analgesics, as they are less stressful.

Study design included a blinded pilot study with a localized perineural injection of human recombinant erythropoietin analog (EPO) at the GEL induced neural site, to observe for an analgesic effect of the EPO on the chronic neuritis in model. This study started on post procedure day 152, followed by behavioral testing on days 153–160.

### Sample size

We tested the null hypothesis ^46^ that the GEL procedure does not differ from the control group during the 5 months after procedure on the dependent variables of paw withdrawals in response to von Frey fibers, a camel-hair brush, and pinprick. The alternative hypothesis was that, over time, there is a difference between the groups. The experiment was designed to discover the smallest biologically important effect, optimizing the number of animals used ^47^.

We conducted a power analysis based on data from previous rat experiments with the GEL model to detect a difference between the GEL and control groups with an unpaired t test if the difference was 1 paw withdrawal and the standard deviation was 0.5 paw withdrawals. We concluded that a minimum sample size of eight per group would yield 95% power with a two-tailed Type I error rate of .05. An additional two to three animals were added to the sham and control groups to compensate for a possible loss of sample size during the 5-month study, and an additional seven animals were added to the GEL group for illness over time, technical complications, and a pilot study with local EPO. GEL *n* = 15, control *n* = 11, sham *n* = 10 were the final initial sample sizes.

### Experimental procedures

***Percutaneous injectable procedure for GEL and sham.*** During isoflurane anesthesia the percutaneous injection procedures were performed. The hydrogel used in the GEL group was the proprietary biological NeuroDigm GEL™, that is composed of purified biocompatible tissue-derived by-products and peptides of mammalian soft tissue, as found in the perineural tissue milieu after a soft tissue injury. Such purified injectable implant products are used in many human procedures and are normally absorbed over days to weeks by the tissues and are rarely antigenic ^48, 49^. A small blade-shaved area (0.6–1.0 cm) on the skin at the distal aspect of the medial Achilles tendon was cleaned with betadine, then 91% alcohol. The skin was then pierced with a sterile 19 gauge needle tip; then a sterile, custom tapered, blunted 21 gauge hollow probe entered the skin puncture site to gain access to the tibial nerve tunnel (U.S. patents 7015371, 7388124). The probe then advanced subcutaneously in a cranial direction; then it pierced the fascia between the distal origins of the medial and lateral gastrocnemius muscle and entered the anatomic tunnel posterior to the tibialis posterior muscle and medial to the soleus, where the tibial nerve courses. Upon entering the neural tunnel, the probe was softly glided in avoiding resistance or nerve contact. In the mid-tibial tunnel of the lower leg 0.3 cc of the GEL™ was implanted or the shams’ Ringer’s lactate was injected, and then the probe was withdrawn, This percutaneous procedure is a refined method ( Supplementary file S3).

***Procedures to elicit neuropathic pain behavior.*** Measures of response to mechanical stimulation were chosen as the dependent variables throughout this entire study. Light touch and pinprick stimulations are commonly used in rodent screening for analgesics and also used in assessing humans. The primary outcome measure was the average number of paw withdrawals to each of five mechanical stimuli applied eight times to each plantar mid hindpaw (tibial n.) of a rat. Each stimulus was applied first to the contralateral hindpaw, then to the ipsilateral side of the GEL and sham procedure. Time between each stimulus application was usually 2–4 seconds or longer. For each stimulus the total number of each hindpaw’s withdrawals was recorded as a data point.

Non-noxious light touch: for static mechanical allodynia von Frey filaments (Semmes Weinstein Mono-filaments North Coast Medical TouchTest®) exerting confirmed forces of 2 grams, 6 grams and 10 grams were used, tips smooth. Forces of fibers were confirmed with a scale to be within 90% of stated force. Dynamic mechanical allodynia was tested with a fan sable brush (09004-1002; Dick Blick Art Materials). The stimuli were applied in the order of: von Frey 2 g, 6 g, 10 g, then brush. Each von Frey stimulus was applied for approximately 1 second until the fiber bent, or the paw was withdrawn. The brush was stroked gently from rear to front of the plantar hindpaw.

Noxious light touch: mechanical hyperalgesia was tested with a custom sharp non-penetrating plastic point calibrated to elicit 2–4 (out of a maximum of eight) paw withdrawals at baseline. This pinprick stimulus tip was touched to the plantar site until the paw withdrew or the skin visibly indented. Each stimulus lasted about 1 second.

***Analgesics administration.*** The analgesics were administered by subcutaneous injection (27 gauge, 1.5”) over the dorsal lower back and proximal thighs, with a custom administrator-held restraint device to reduce handling and stress. Morphine sulfate (West-Ward) was mixed with normal saline and administered at a dose of 3 mg/kg 1 hour prior to screening the analgesic. The vehicle used in mixing the following three drugs was 0.25% methylcellulose *(*Methocel ^®^ A4M Premium LV USP). These three drugs were mixed 24–48 hours prior to use. Celecoxib (Cayman Chemical) was dispensed at a dose of 10 mg/kg 1 hour prior to screening; gabapentin (Cayman Chemical) was dispensed at a dose of 25 mg/kg 2 hours prior to screening; and duloxetine (Cayman Chemical) was mixed (mechanically agitated) and administered at a dose of 10 mg/kg 2 hours prior to screening. The experimenter knew the drugs being screened; the group identity of the rats was blinded throughout experiment. The injected volume of each drug was less than 1.2 cc.

The original doses chosen for gabapentin and duloxetine had marked adverse effects in this study of aged mature rats, interfering with the testing of pain behaviors. Gabapentin at 60 mg/kg had marked ataxia in all rats, with their hindpaws not staying on testing screen due to lumbering gait and falls. Duloxetine at 30 mg/kg was noted for marked “frozen” hypoactive posture, with increased tone and alertness (no central sedation) to normal handling and testing. After duloxetine was given at this dose, paw withdrawals were not elicited in any of the three groups. These adverse effects interfered with drug screening. Lower doses of gabapentin 25 mg/kg and duloxetine 10 mg/kg were used in the study, without adverse drug side effects and improved the ability to test paw withdrawals ^50^.

### Erythropoietin treatment pilot study

Epoetin alfa (EPO) by Amgen, a recombinant human erythropoietin analog at 2000 units/mL, was diluted 1:3 with normal saline, and 0.3 cc vol., 200 units, was the administered dose in the pilot study. After the main experiment was over the 14 GEL procedure rats continued in a pilot study for 8 days beginning on day 152, with days 140, 149 and 152 taken as baseline days. Three subgroups were picked randomly (same as prior method of simple randomization) and the experimenter was blinded during screenings for pain behavior. On day 152 under isoflurane, as described prior, the “EPO at site” group ( *n* = 5) received an injection of 200 units of EPO as a perineural infiltration at the site of the original GEL procedure on the ipsilateral leg. The “EPO SC” group ( *n* = 4) received the same EPO injection subcutaneously on the dorsal low back, and the “No EPO” group ( *n* = 5) received no injection. The original “EPO at site” injection approach was ipsilateral (left) posterior-to-anterior at mid tibia through the bellies of the gastrocnemius muscle aiming for the tibial nerve tunnel. Pinprick behavior data were collected on days 153, 154, 156, 159, and 160.

Two of the five “EPO at site” rats had no decrease in paw withdrawals with the original technique of the EPO injection on post procedure day 152. To improve localization, on post day 155 these two rats had an adapted lateral approach of the injection of 200 units of EPO near the original ipsilateral GEL™ procedure site. This adapted injection was through the lateral gastrocnemius muscle targeted to the mid tibial tunnel at lower leg.

### Histology

At the conclusion of the study, three rats were chosen randomly from each of the three groups: 1.) GEL procedure rats 2.) sham procedure rats of the 5/8 that displayed late onset robust pain behavior, and 3.) controls. The selection from the GEL group contained two rats that were controls in the EPO pilot study, and one that had received the subcutaneous EPO injection. The animals were anesthetised, and then perfused with Lactated Ringer’s solution (Hospira), followed by perfusion fixation with 4% paraformaldehyde (PCCA Professional Compounding Centers of America) in Phosphate Buffered Saline (PBS) (Electron Microscopy Services). Following fixation, the lower limb on the ipsilateral side was grossly dissected to reveal the gastrocnemius muscle thus providing a landmark for locating the tibial nerve. Once identified, the distal tibial nerve (below the popliteal area) was dissected free of the surrounding muscle and fascia, and placed into ice-cold 4% paraformaldehyde in PBS for overnight incubation. The following day the paraformaldehyde solution was replaced with 30% sucrose (IBI Scientific) to cryoprotect the tissue. The cryoprotected samples were embedded in Tissue-Tek OCT (Sakura Finetechnical, Japan) and frozen on dry ice. Cryosections (10 μm) were then prepared and mounted onto SuperFrost Plus slides (Fisher Scientific, Rockford, IL). Sections were then fixed in 10% Neutral Buffered Formalin for 10 minutes, washed for 5 minutes in 1X PBS to remove OCT, and rinsed with tap H _2_O. Subsequently, sections were then stained in Hematoxylin (Fisher Scientific) for 5 minutes and rinsed with tap H _2_O, differentiated in acid alcohol (1% HCl in 70% EtOH) for 30 seconds and rinsed extensively with tap H _2_O, blued in 0.2% aqueous ammonia, rinsed with tap H _2_O, and stained with eosin (Fisher Scientific) for 1 minute. Sections were then dehydrated by sequential submersion in graded 75%, 95%, 100% EtOH for 5 minutes each, and a final submersion in xylene. The slides were air dried before mounting with Permount (Fisher Scientific) and adding coverslip. Sections were viewed and the images captured on a Nikon 80i microscope, outfitted for digital light micrographs.

### Statistical methods

***Pain behavior statistical analyses.*** As described in detail in the results section, the data were inspected for compliance with the assumptions of ANOVA. Two areas of concern were noted, particularly the heterogeneous variances in the pinprick data and the very large number of pairwise comparisons that could be compared. The former occurred because (a.) GEL animals that developed pain symptoms tended to eventually score the maximum number of 8 withdrawal responses, leading to some cells with very small or zero variance in the GEL group only, and (b.) the animals in the sham group were not homogeneous in their response to the sham procedure and this greatly increased their variance. We proceeded with the ANOVA for pinprick because of the convenience of describing interaction effects and for comparison with the allodynia data. We note that the pinprick variable was the least likely to generate errors of inference because of the very large effects and consequent minuscule *p* values obtained. Individual sham data are plotted in a separate graph to illustrate the problem there. Type I errors were reduced by testing only planned comparisons among a relatively small number of means and by combining data where appropriate before analysis so that fewer comparisons would be made.

Analyses of the paw withdrawals in response to von Frey fibers, the brush, or the pinprick on the routine test days were conducted using a mixed model ANOVA with one between groups factor (eleven controls, fourteen GELs, and eight shams) and two repeated measures factors. The first repeated measures factor was the time the data were collected, with an average baseline period of 4 days prior to the procedures and the five 30-day periods, referred to as post procedure Monthly Periods 1 through 5 (P1, P2, P3, P4, P5), following the procedure ( Figure 1). For analyses, the data point for each animal in each monthly period was the mean of at least four routine pain behavior testing days during that month. The second repeated measures factor for the von Frey fiber analysis was a composite factor combining the three levels of fiber force (V1, V2, V3) and the two sides for a total of six samples of different forces tested on bilateral hindpaws. Differences owing to fiber force and sidedness were determined by comparing means with planned comparisons. The second repeated measures factor for the brush and pinprick were the bilateral hindpaws. In the global ANOVA, a *p* value of < .05 was considered significant. Except for the von Frey analysis, planned comparisons were conducted using Fisher‘s Least Significant Difference test after a global ANOVA was determined to be significant at the .05 level with a two-tailed test (Dataset was used in all analyses).

***Analgesic statistical analyses.*** Experiments were conducted with four analgesic drugs administered shortly before the usual testing with von Frey fibers, the brush, and the pinprick. The four drugs were each tested three times during the post procedure period from day 28 to day 149. The effects of the analgesic drugs were analyzed on two dependent variables instead of five (allodynia measures averaged together as one variable and the hyperalgesia measure of pinprick as the other). The data were analyzed using a mixed model ANOVA with the three groups as a between-subjects factor and side (left or right) and days (three pairs of pre-drug and analgesic drug days) as repeated measures factors. The effect of the analgesic drug for each pair of days was analyzed using planned comparisons. These comparisons used Fisher’s Protected Least Significant Difference test if the corresponding *F*-ratio was significant or used a Bonferroni-protected contrast if the *F*-ratio was not significant. All tests used a two-tailed significance level of .05.

## Results

### Clinical observations

All rats had recovered from anesthesia within 5 minutes and were walking normally without altered gait. Following recovery from anesthesia the subjects did not demonstrate observable pain behaviors ^51^ nor clinical evidence of tissue injury. Throughout the duration of the study all the rats were observed to have normal gait and were without visible evidence of inflammation, swelling, increased warmth, weakness, deformities or positional changes noted on the operated hindpaw, at any time. Specifically, there was no observed evidence of acute nociceptive pain after the procedures for 2 weeks. Acute inflammation and neuropathic pain can be manifested by hypersensitivity such as allodynia and hyperalgesia ^52^, yet no such abnormal hypersensitivity was present in the four testing periods of the first 14 days. No other evidence of discomfort or pain in the rats including aggression, loss of appetite, abnormal posturing, porphyrin secretion, piloerection, or decreased activity ^53^ was present. Their grooming activities were normal.

Among the GEL group, 14/14 rats had markedly increased paw withdrawals to pinprick, von Frey fibers and brush by day 23 post procedure. The paw withdrawals to pinprick became more exaggerated over the remaining months. By post procedure day 60, 5/8 of the sham rats had developed marked paw withdrawals to pinprick. The control group’s behavior never changed. The most common pain behavior was a sudden reflexive paw withdrawal reaction. Other reactions appearing one month after increased pain behaviors included prolonged shaking and or licking of their affected ipsilateral paw. Similar patterns of paw withdrawal reactions occurred on testing the contralateral side as pain behavior developed 2–3 weeks after the ipsilateral hindpaw. No biting or chewing of the paws occurred.

Results are presented below with the statistics using Dataset 1; also see Supplementary file S4: Basic Anova statistics, for a comprehensive description of the pain behavior statistical results without drugs.

Raw data of NeuroDigm Model of neuropathic pain in mature rat The raw data used for the statistical studies are provided. Click here for additional data file. Copyright: © 2017 Hannaman MR et al. 2017 Data associated with the article are available under the terms of the Creative Commons Zero "No rights reserved" data waiver (CC0 1.0 Public domain dedication).

### Results of behavioral data

***Pain behavior analyses.*** While the hyperalgesia in this mature GEL™ model was robust over time, the allodynia was a minor response. Before attempting a statistical analysis, we plotted the raw routine screening day’s data (without analgesics) of each group for inspection. The robust effects of pinprick hyperalgesia were evident in the raw data graph. The small effects observed with the data of each individual allodynia stimulus (each of three von Frey fibers and the brush) indicated that an individual routine screening day’s mean in the GEL group could not reliably be expected to differ from the control group. We were able to achieve this reliability by grouping the data in two ways:

First, *different days* of testing for each stimulus could be averaged together for an individual variable, such as data for each individual von Frey fiber averaged together over 4 testing days to make a monthly average: this method was used in the allodynia and hyperalgesia line graphs ( Figure 3– Figure 5).

Second, the scores for *all four allodynia measurements* could be averaged together to make a summary single variable, i.e., averaging data for all three von Frey fibers and the brush into a single number representing allodynia. This method was used for allodynia in the analgesic drug response bar graphs, to compare one day of pre-drug data to one day of post-drug data ( Figure 7– Figure 10).

These methods have different advantages. Plotting each day’s data is useful for determining the precise timing of when effects emerge during the long period of testing. Plotting monthly data is useful for observing the small effects that are apparent between the individual von Frey fibers. Information about effect sizes will be presented for the routine screening day’s data of the averaged variable (von Frey plus brush), and formal statistical analysis will be applied to the much smaller number of means in the monthly data for each individual variable.

**Figure 2.**
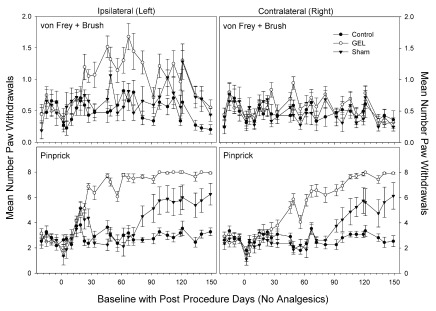
Test days without analgesics; average of all allodynia responses (top) and average of pinprick responses (bottom). The averaged allodynia measures on the ipsilateral side were more consistently different on a routine test day basis than any of the individual allodynia measures (top: y-axis is 2, maximum is 8; bottom: maximum y-axis is 8). Shams are not homogenous: 5/8 with pinprick hyperalgesia, 3/8 similar to controls. GEL n = 14, sham n = 8, control n = ll.

***Days of data pattern of allodynia and hyperalgesia after GEL procedure.*** The data for all routine testing days (no analgesics given) for the combined allodynia variable (von Frey plus brush, top) and for the hyperalgesia variable (pinprick, bottom) are presented in Figure 2 below. The most noticeable effect is the increase of pain behaviors in response to the pinprick in the GEL group on both the ipsilateral and contralateral sides. The pinprick hyperalgesia effect occurred in every rat subjected to the GEL procedure. Although the pinprick responses required about 23 days to develop, the symptoms, and therefore the opportunity to study those symptoms, persisted for months and showed no sign of waning by the end of the experiment. Under conditions of a null effect, each of two groups would be expected to have a greater mean than the other about 50% of the time. However, the last day on which the control group had an absolutely greater mean was for pinprick post procedure day 5 on the ipsilateral side and day 23 on the contralateral side.

Table 1 provides estimates of effect sizes for the GEL™ effect, which tends to increase with time. The standardized effect size was calculated as the difference between the means of the GEL and control groups on that day divided by the pooled standard deviation of the two groups. These can be used to plan future experiments depending on which interval after the procedure will be studied. Minimum sample sizes are provided to yield at least 80% power in a two-group, two-sided *t*-test with a Type I error rate of .05. Larger sample sizes are required to study allodynia than to study hyperalgesia. Designs more complex than a simple *t*-test, such as the present study, which include many repeated measurements and multiple groups, will have more error degrees of freedom for the comparisons than a simple *t*-test, and will not require such large sample sizes for the allodynia measures.

**Table 1.** Effect size (ES) data for hyperalgesia and allodynia on routine test days for control (C) and GEL (G) groups.

Effect	Ipsilateral	Contralateral	Calculated n
**Hyperalgesia (Pinprick)**			
Last day C > G	5	23	NA
ES > 1.94 SDs	day 23–149	day 72–149	6
ES > 2.83 SDs	day 46–149	day 92–149	4
ES > 3.50 SDs	day 72–149	day 106–149	3
**Allodynia (von Frey plus brush)**			
Last day C > G	19	149	NA
ES > 0.71 SDs	day 23–149	--	32
ES > 0.86 SDs	day 112–149	--	22
ES > 0.91 SDs	day 119–149	--	20

*Included are the last day that the control group mean exceeded the GEL group mean, and the inclusive days that the size of effect for the GEL group exceeded the control group by 1.94, 2.83, or 3.50 standard deviations (SDs). ES is the difference between the group means divided by the pooled standard deviation of the two groups. The calculated n is the sample size required to detect a difference between GEL and control groups of the given size for the hyperalgesia or allodynia variable on a routine test day basis in an independent-samples t-test with 80% power and a two-sided alpha of .05. Allodynia was not consistent on the contralateral side. See routine test day means in Figure 2. NA, not applicable.*

Using these data, analgesic screening can start on day 23 or later with a group of *n* = 6 rats using pinprick (mechanical hyperalgesia) on the ipsilateral side. The effect size for hyperalgesia on the ipsilateral side was persistent at more than 1.94 standard deviations from day 23 until the end of the main experiment on day 149. Smaller effects of the GEL group were observed for the combined allodynia variable (von Frey plus brush) than for hyperalgesia with the pinprick. Unlike the data for the individual von Frey and brush variables, the combined variable shows clear and persistent differences between the GEL and control groups on the ipsilateral side for each testing day. This is important for our subsequent experiments with analgesics. The effect of the GEL™ procedure is not constant across time. Therefore, when testing the effect of an analgesic on a single day with a control value from the same animal, the response must be compared to a control day very close in time to the day the analgesic is given rather than to the average of all control days. To do this successfully, each control day must show a positive effect of the GEL procedure, and this was not true on every day for the individual von Frey fibers or the brush alone. Consequently, we opted to use the combined von Frey plus brush data for all comparisons between individual analgesic and control days (see the section, *Results of experiments with analgesic drugs,* where statistical analyses of those days are provided). 

In order to provide a formal statistical analysis of the individual allodynia variables, we averaged all days (at least 4) within each month that the animals were tested without analgesics to remove some of the test day variability, stabilize the means, and estimate effect sizes. These analyses are presented in the following sections.

**Figure 3.**
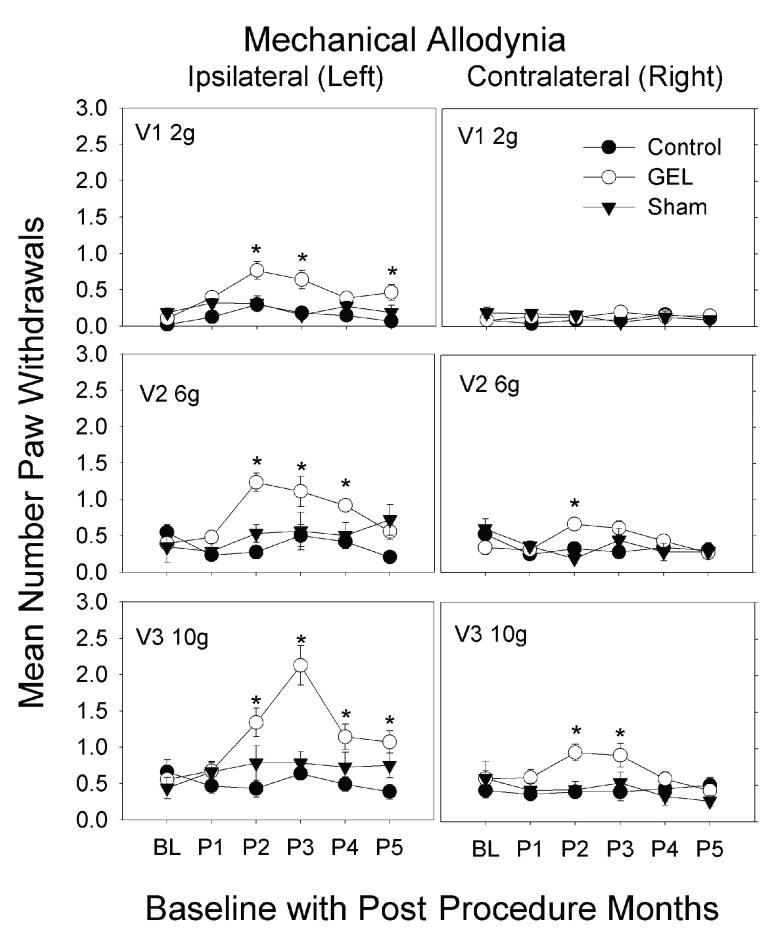
Mechanical allodynia to light touch over 5 months. These graphs depict von Frey fiber results for paw withdrawals ipsilateral and contralateral, for three fiber forces over 5 monthly post procedure periods (P1–P5) (maximum y-axis is 8). Mean and S.E.M. * *p* < .05 GEL group greater than both GEL group baseline and control group for same period. GEL n = 14, sham n = 8, control n = ll.

***Mechanical allodynia: von Frey analysis.*** The GEL group showed prolonged pain behavior of mechanical allodynia to von Frey fiber stimuli with increased paw withdrawals, bilaterally after 1 ^st^ month, except for the contralateral 2 gram fiber. This pain behavior peaked by the 2 ^nd^ to 3 ^rd^ month then waned, returning to near baseline by the 5 ^th^ month. The data are given in Figure 3 for the three different fiber forces in each group, over all periods applied to both the ipsilateral and contralateral sides. The highest-order interaction of the ANOVA was significant ( *F* (50, 750) = 2.21, *p* < .001). The sham and control groups never significantly exceeded their respective baseline value in any monthly period on either side.

**Figure 4.**
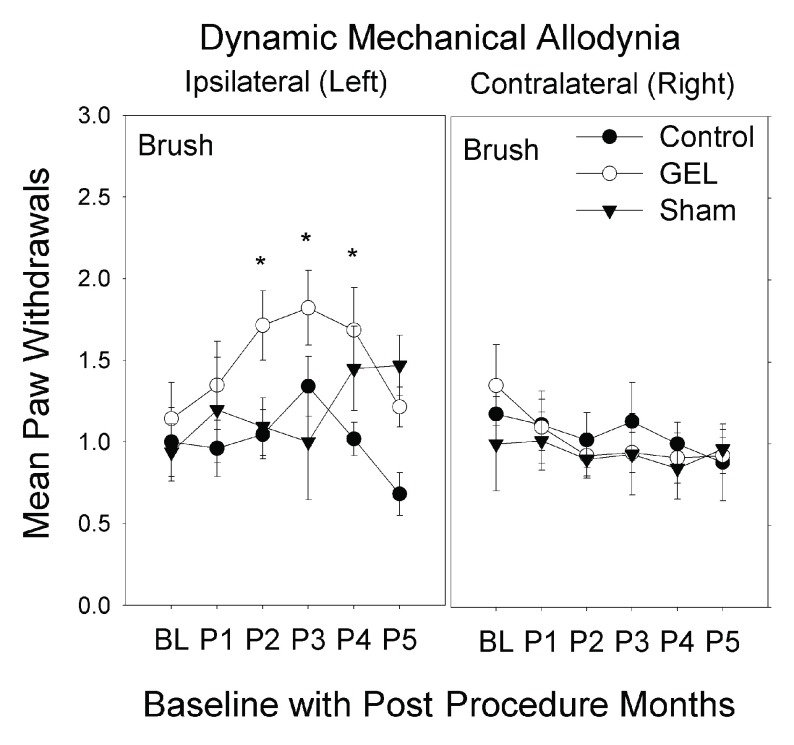
Dynamic mechanical allodynia to brush over 5 months. These graphs depict brush results for paw withdrawals ipsilateral and contralateral, over 5 monthly post procedure periods (P1–P5) (maximum y-axis is 8). Mean and S.E.M. * *p* < .05 GEL group greater than both GEL group baseline and control group for same period. Reduced responding on the ipsilateral side in the GEL and control groups during period 5 may reflect habituation, which is not present in the shams as their allodynia increased in P4–P5. GEL n = 14, sham n = 8, control n = ll.

***Mechanical allodynia: brush analysis.*** The GEL group showed prolonged pain behavior of dynamic mechanical allodynia to brush stimuli with increased paw withdrawals, only on the ipsilateral side after the 1 ^st^ month. This pain behavior peaked by the 3 ^rd^ month then waned, returning to near baseline by the 5 ^th^ month. The shams had similar pain behavior on the ipsilateral side that plateaued by the 4 ^th^ month, persisting until the 5 ^th^ month at end of study ( Figure 4). The highest-order interaction was significant ( *F* (10, 150) = 1.943, *p* = .044). The control group never significantly exceeded the baseline value in any monthly period on either side.

***Mechanical hyperalgesia: pinprick analysis.*** The GEL group had the earliest and most persistent pain behavior of mechanical hyperalgesia with increased paw withdrawals to pinprick, bilaterally. The hyperalgesia was first present on the left side and within a few weeks present on the right side. This pain behavior was vigorous after the first month and persisted robustly for 4 months, until the end of the study. Many of the shams had similar pinprick pain behavior bilaterally that peaked by the 4 ^th^ month and persisted until the 5 ^th^ month, at end of study. The control group had no pinprick pain behavior during the study.

**Figure 5.**
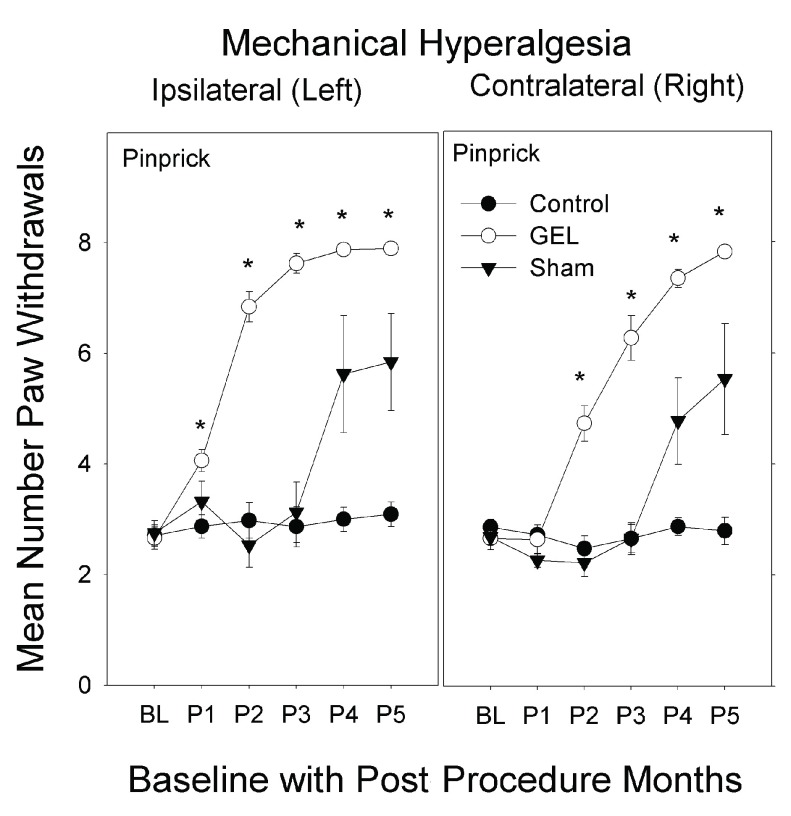
Mechanical hyperalgesia to pinprick over 5 months. In the GEL group, 2–3 weeks following the onset of hyperalgesia on the left it began to develop on the contralateral side; a similar slight delay in the contralateral onset of hyperalgesia is seen in the shams. The graphs depict paw withdrawal responses to pinprick on the ipsilateral and contralateral sides during the 5 monthly post procedure periods (maximum y-axis is 8). Mean and S.E.M. *p < .05 GEL group greater than both GEL group baseline and control group during the same period. GEL n = 14, sham n = 8, control n = ll.

The data for pinprick are presented in Figure 5. The highest-order interaction was significant ( *F* (10, 150) = 4.592, *p* < .001). The control group never deviated from its own baseline value in any post procedure period on either side. By contrast, the GEL group’s paw withdrawal response on the ipsilateral side was significantly greater than baseline during all five post procedure periods, and the response on the contralateral side was significantly greater than baseline during periods 2 through 5. Between-group comparisons for pinprick indicated that the three groups were not significantly different during the baseline period on either the contralateral or ipsilateral side. 

**Figure 6.**
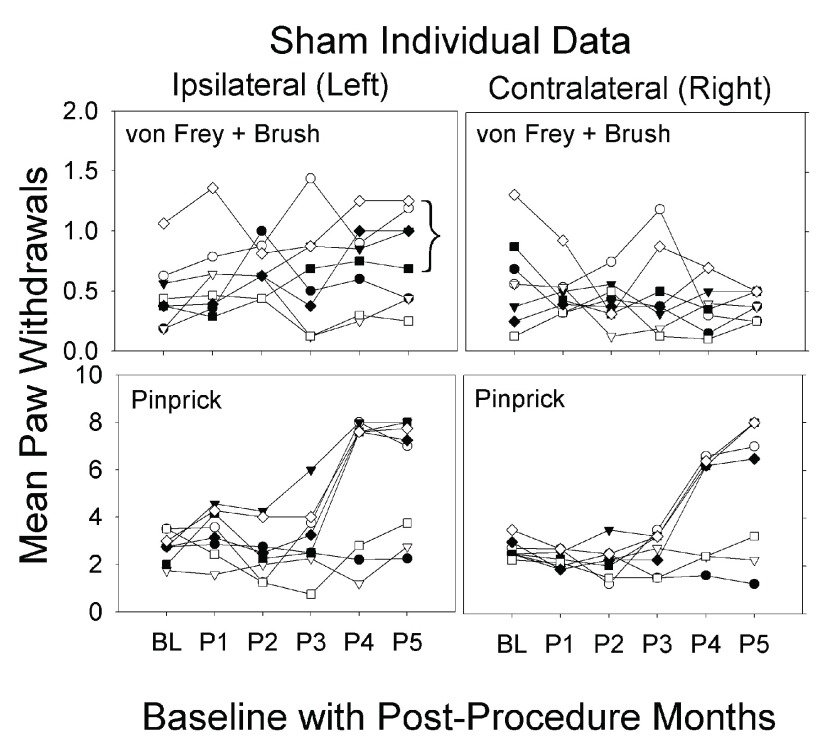
Individual sham data over 5 months. Graphs depict average of paw withdrawals for eight individual shams. Maximum on y-axis is 8 for von Frey and pinprick. Bracket in allodynia graph identifies the same five responders from the hyperalgesia data. Sham n = 8.

***Individual data for sham group.*** Retrospectively, we noted that five of eight sham procedure animals developed pain behavior bilaterally, similar to the GEL™ animals, in post procedure monthly periods 4 and 5 (after 3 months); and the three remaining sham rats behaved similarly to the control group. Due to the behavioral divergence in this group after three months, statistical analysis was not performed for any difference between the GEL and sham groups over time. The reason for this is that the sham group itself was not homogeneous in the responses that developed after the sham procedure. Individual data for the sham animals are presented in Figure 6. The data on the shams, in all the pain behavioral studies and in the analgesic screening, included the results from all eight shams. The probability that the bracketed five responder sham rats would separate themselves from the other three in exactly the same direction by chance in the allodynia experiment is .017 for a single day. This probability does not factor in the magnitude of the effect between responders and non-responders or the fact that they separated themselves the same way on the same two consecutive days as in the hyperalgesia experiment. This is very strong evidence that we detected ipsilateral allodynia in all of the same sham animals where we detected hyperalgesia.

***Factor influencing pain behavior testing.*** A reportable factor during this study relates to the influence of hormone replacement of the experimenter on the elicited responses to stimuli. In nine early environmental influence screenings (see study design) paw withdrawals were markedly less to nonexistent bilaterally in the first random six rats screened as compared to their prior screening session. Thirty minutes after the topical application to the investigator of a 17 beta-estradiol replacement cream, the screening was repeated on the same six rats, and their elicited pain behaviors were then consistent with the data collected during the prior testing period. On unblinding, this effect was not related to groups. Even baseline pre-procedure behaviors were similarly affected by the estrogen hormone replacement. All data used from this study was collected with topical estrogen applied 30 minutes prior to beginning all testing.

### Results of experiments with analgesic drugs

All animals in the each of the three groups GEL (n = 14), sham (n = 8), and control (n = 11) were screened with the same subcutaneous drug and dose on each day of testing with an analgesic. To control for the effects of time, it was important to compare the data for each analgesic’s screening day to a single pre-drug control day, prior to the drug’s administration. As illustrated in the top part of Figure 2, an effect on the GEL group’s ipsilateral side was apparent on individual control days when the paw withdrawals for all four allodynia measures were averaged together into one variable. For that reason, we analyzed only the composite allodynia variable and the pinprick for responses to the analgesics. The data for each analgesic drug are displayed in the figures as bars for the mean response, with the analgesic drug on a particular day (the days listed in the x-axis label) paired with the respective control data from the routine test day one to four days prior. Throughout the following analyses, the mean number of paw withdrawals in the GEL group on the ipsilateral side was significantly greater than that of the control group on all pre-drug days for both the von Frey plus brush variable and for the pinprick.

**Figure 7.**
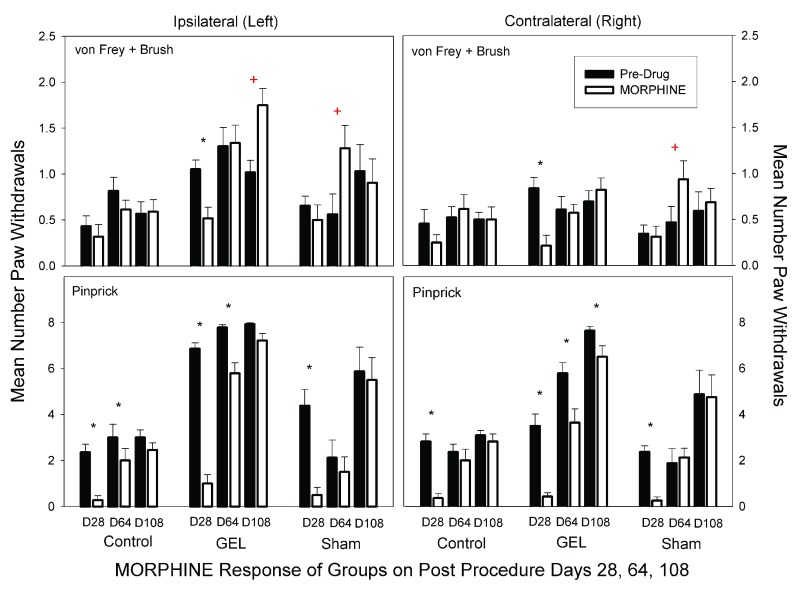
Morphine analgesia results. Morphine reduced paw withdrawals less over time. The graph depicts the average of paw withdrawals on pre-drug control days (black) and on paired morphine dose days (white). Results of behavior testing show the average of all four light touch allodynia measures (three von Frey fibers and the brush, top graphs) and the mechanical hyperalgesia (pinprick, bottom graphs). Mean and S.E.M. *Significant decrease from the paired control day, *p* < .05. + Significant increase from the paired control day, *p* < .005. GEL n = 14, sham n = 8, control n = ll.

***Morphine.*** Morphine sulfate at 3 mg/kg had an escalating loss of effectiveness bilaterally. Morphine at 3 mg/kg is usually a toxic dose in humans. The data for the morphine test days using the von Frey fibers and brush are presented in the top half of Figure 7. The ANOVA revealed that all main and interaction effects were significant at the .05 level including the three-way interaction ( *F* (10, 150) = 2.322, *p* = .014). Morphine caused significant decreases in responding compared with pre-drug day on both the ipsilateral and contralateral side only on day 28. In three instances, morphine actually increased pain responses, as denoted by red plus symbols. These were the only significant increases in pain behavior resulting after an analgesic in the entire dataset for the four analgesic drugs. The data for pinprick are presented in the bottom half of Figure 7. The ANOVA revealed that all main and interaction effects were significant including the three-way interaction ( *F* (10, 150) = 2.655, *p* = .005). The shams and controls also suggest an escalating pattern of stimuli sensitivity after morphine with pinprick.

Morphine was effective early after the GEL™ procedure, but the size of the effect waned with time on both sides. For example, in the GEL group on the ipsilateral side for the allodynia measures, the standardized effect size between the control day and the morphine day changed from a positive analgesic effect of 1.30 pooled standard deviation units on day 28 to no effect on day 64 to a negative effect of -1.25 pooled standard deviation units on day 108. For the pinprick measure, effect sizes were conservatively estimated using the standard deviation for the morphine condition only instead of the pooled standard deviation because of the reduction of the variability as the responses approached a ceiling of eight paw withdrawals out of eight pinpricks. The analgesic effect size on the ipsilateral side with pinprick waned from 4.14 standard deviations on day 28 to 0.64 standard deviations on day 108.

**Figure 8.**
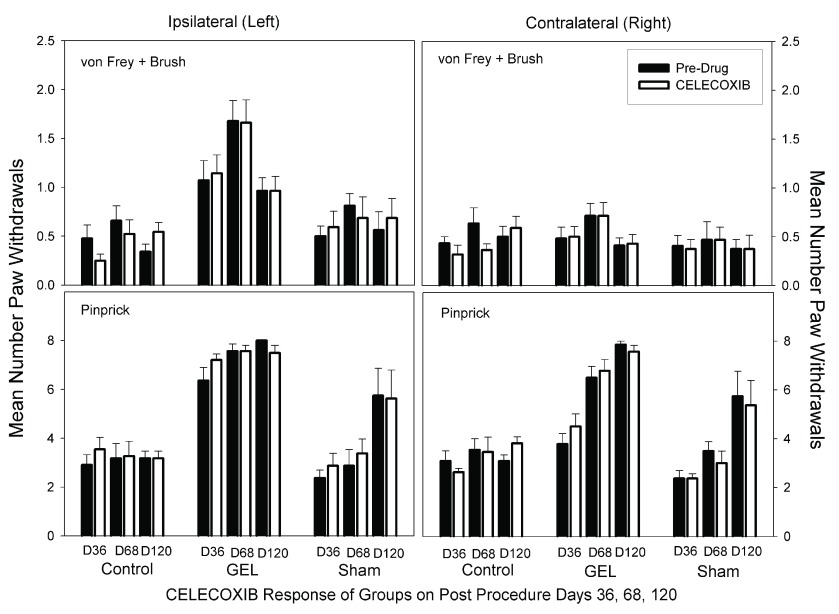
Celecoxib analgesia results. Graph depicts average of paw withdrawals on pre-drug control days (black) and on paired celecoxib dose days (white). Results of behavior testing show the average of all four light touch allodynia measures (three von Frey fibers and the brush, top graphs) and the mechanical hyperalgesia (pinprick, bottom graphs). Mean and S.E.M. Celecoxib did not significantly reduce paw withdrawal responses on any pair of days for any groups. GEL n = 14, sham n = 8, control n = ll.

***Celecoxib.*** There was no analgesic effect of celecoxib at 10 mg/kg in any group on any day on either side. Celecoxib did not decrease pain behaviors, as demonstrated with a decrease in paw withdrawals. Celecoxib at 10 mg/kg is about three times a human dose. Data for the celecoxib days for the von Frey fibers and brush are presented in the top half of Figure 8. The ANOVA revealed significant main effects of groups, side, and days, and a significant group-by-side interaction ( *F* (2, 30) = 22.92, *p* < .001). None of the other interaction effects was significant. There was no effect of the celecoxib dose on either side in any group by Bonferroni-protected planned contrasts. The data for pinprick are presented in the bottom half of Figure 8. The ANOVA revealed that all main and interaction effects were significant, including the three-way interaction ( *F* (10, 150) = 2.675, *p* = .005. The significance of this interaction was completely accounted for by other effects in the data that were not related to any specific pre-drug vs. drug contrast in our set of planned comparisons.

**Figure 9.**
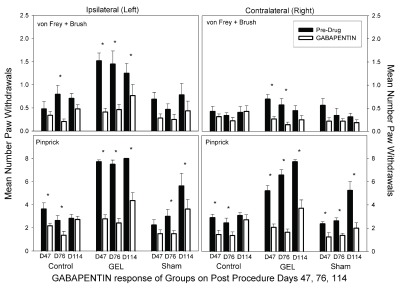
Gabapentin analgesia results. Graph depicts average of paw withdrawals on pre-drug control days (black) and on paired gabapentin dose days (white). Results of behavior testing show the average of all four light touch allodynia measures (three von Frey fibers and the brush, top graphs) and the mechanical hyperalgesia (pinprick, bottom graphs). Mean and S.E.M. *Significant decrease from the paired control day, *p* < .05. Gabapentin also suppressed responding in the sham and control group. GEL n = 14, sham n = 8, control n = ll.

***Gabapentin.*** On all days on both sides in the GEL group, gabapentin at 25 mg/kg robustly reduced paw withdrawal responses ( Figure 9). The dose of gabapentin used is nearly equivalent to a human dose. Gabapentin significantly reduced the sham group’s pinprick responses on most days. Gabapentin also significantly reduced the level of responding in the control group on days 47 and 76 on both sides. The data for the gabapentin test days using the von Frey fibers and brush are presented in the top half of Figure 9. The ANOVA revealed significant main effects and interaction effects except for the three-way interaction. It appears that the group-by-days pattern of responding was similar on the ipsilateral and contralateral sides, therefore, the groups-by-days interaction term is the important one for analysis ( *F* (10, 150) = 1.929, *p* = .045). When the data for the ipsilateral and contralateral sides were combined, we found that gabapentin robustly reduced the GEL group responding on all analgesic test days. For comparison to other figures, asterisks in the top half of Figure 9 represent significant decreases from the paired pre-drug day by Bonferroni-protected planned contrasts. By this analysis, the comparison for day 114 on the contralateral side for the allodynia measure in the GEL group was not significant. The data for pinprick are presented in the bottom half of Figure 9. With the exception of the group-by-side interaction ( *p* = .055), all main and interaction effects were significant at *p* < .05 including the three-way interaction ( *F* (10, 150) = .029, *p* = .03).

**Figure 10.**
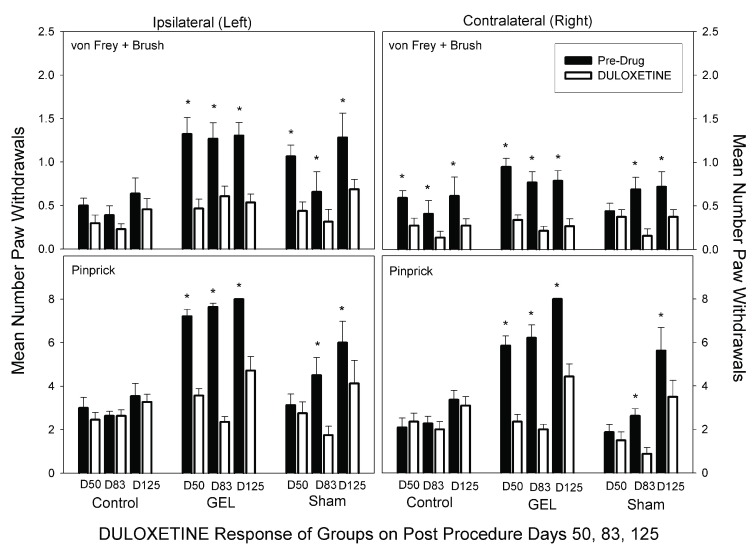
Duloxetine analgesia results. Graph depicts average of paw withdrawals on pre-drug control days (black) and on paired duloxetine dose days (white). Results of behavior testing show the average of all four light touch allodynia measures (three von Frey fibers and the brush, top graphs) and the mechanical hyperalgesia (pinprick, bottom graphs). Mean and S.E.M. *Significant decrease from the paired control day, *p* < .05. Duloxetine had less effect than gabapentin on the responses in the control group. GEL n = 14, sham n = 8, control n = ll.

***Duloxetine.*** Duloxetine at 10 mg/kg reduced pain behaviors bilaterally in the GEL group (Bonferroni-protected contrasts). This dose is markedly less than most prior rat doses ^50^, and more than human doses. The bilateral analgesia was similar in the sham group on D83 and D125, but emerged only after the pain behaviors began developing after 2 months. Interestingly, duloxetine did not suppress normal responses to pinprick stimuli in the control group as the gabapentin did. Yet the duloxetine did suppress responses of the contralateral allodynia in the control group. The von Frey fibers and brush data for duloxetine are presented in the top half of Figure 10. The ANOVA revealed a significant three-way interaction ( *F* (10, 150) = 1.99, *p* = .039). The data for pinprick are presented in the bottom half of Figure 10. The ANOVA revealed that all main and interaction effects were significant except for the three-way interaction. The groups-by-days interaction was significant ( *F* (10, 150) = 11.358, *p* < .001), and the pattern of responding within the groups was similar on the ipsilateral and contralateral sides.

### Reversal of GEL effect with EPO

**Figure 11.**
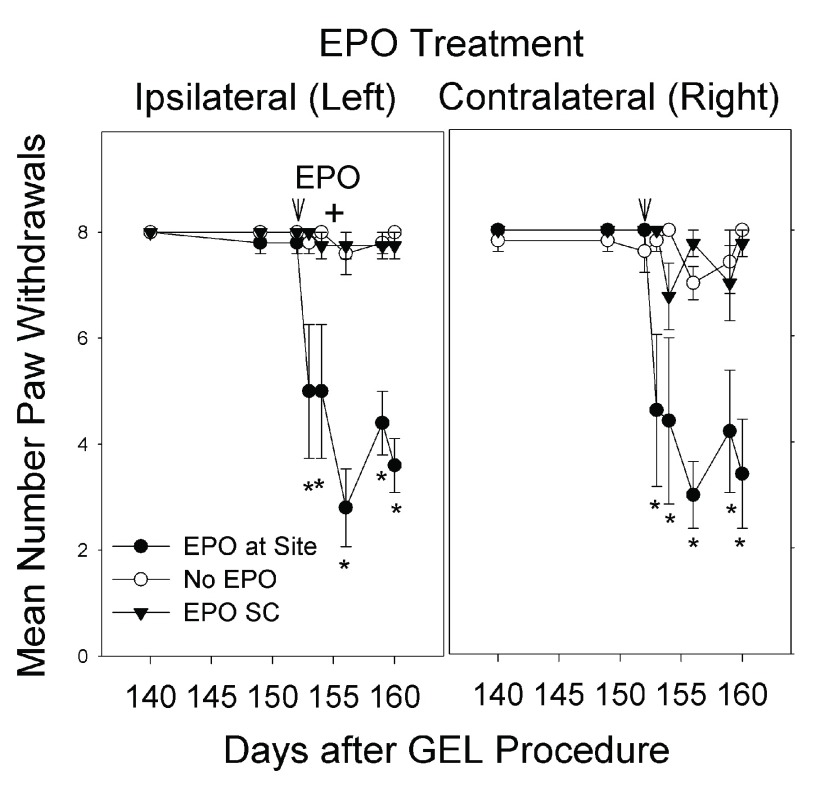
Pain behavior reversed after EPO was injected at the ipsilateral GEL site. After a perineural infiltration of EPO (200 units in NS) the paw withdrawals to pinprick decreased bilaterally to near pre-GEL procedure levels. The groups of “No EPO” control and “EPO SC” injected subcutaneously continued with robust pain behavior, bilaterally. The **+** on day 155 signifies 2/5 rats in the “EPO at site” group re-injected with EPO to ensure accurate localization. * *p* < .001 “EPO at site” vs. “No EPO”. EPO at site n = 5, No EPO group n = 5, EPO subcutaneous group n = 4.

The pain behavior in the GEL group that had persisted for 4 months was reversed for up to 7 days (end pilot), by the targeted perineural application of epoetin alfa (EPO) in a pilot study, at the end of the investigation ( Figure 11). Two of the rats in the "EPO at site" group received a second local EPO injection with a lateral approach on day 155 (seen as a + sign on the left paw results in Figure 11), described in the Materials and Methods section on Erythropoietin treatment pilot study. Pinprick behavior data were collected on days 153, 154, 156, 159, and 160. The resulting data were analyzed using a mixed model ANOVA, with the “ *EPO at site”* injection group as the *between subjects* factor (three groups), and *days* as one repeated measures factor (8 days), and *laterality* as a second repeated measures factor. The three-way interaction was not significant, but the groups-by-days interaction was significant ( *F* (14, 77) = 8.208, *p* < .001). As can be seen in the graph, the effect of EPO was nearly identical on the right and left sides and was significant for at least 6 days, end of study. An ANOVA such as this should be interpreted with caution because of several cells with zero variance (there is a ceiling effect of eight paw withdrawals out of eight stimulus presentations of pinprick). A typical post-hoc power analysis was problematic because of the heterogeneous variances. Instead, we simulated the experiment 100,000 times using the last day’s means and standard deviations on the ipsilateral side as population values to get an estimate of power ^46^. The null hypothesis was rejected at the .001 level over 99% of the time indicating very high power. As an alternative analysis, we conducted a nonparametric Kruskal-Wallis test on the last day’s data with the result H (2) = 11.27, p = 0.004, which is remarkable with small sample sizes, given that the test does not weight the magnitude of the effect.

### Results of histology 

**Figure 12.**
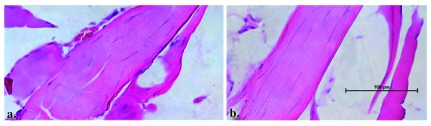
Normal tibial nerve histology in both the control and sham procedure groups. Longitudinal sections through the control ( **a**.) and sham ( **b**.) procedure groups reveal normal appearing nerves. The surrounding muscle tissue that was harvested with the nerve also appears to be within normal limits.

 The tissue sections from nine rats randomly selected from all groups were blinded, and observed by an independent neuropathologist. These observations were later respectively matched to each of the three groups, with *n* = 3 GEL procedure rats, *n* = 3 sham procedure rats of the 5/8 that displayed late onset robust pain behavior, and *n* = 3 controls. Details are described in *Histology* within the Materials and Methods section. There were no differences or abnormal findings noted in the tissue sections between the control and the sham procedure animals. The structure of the nerve and surrounding tissue was completely unremarkable ( Figure 12).

**Figure 13.**
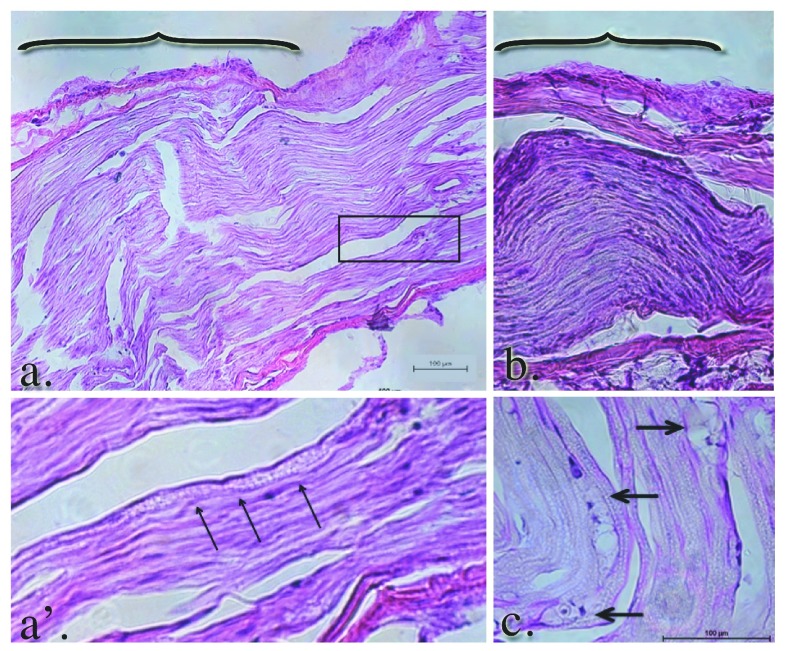
Edema and Wallerian degeneration in the nerves of GEL procedure rats. Panels **a**. and **b**. reflect the gross swelling seen in the tibial nerves upon dissection. The brackets in panels **a**. and **b**. denote the prominent areas of swelling in the GEL nerves. The axons within the swollen area are themselves swollen. The diameters of the axons in this distended area of the nerve are approximately twice the diameter of the contiguous axons proximal or distal (not shown) to it. The arrowheads point to axonal debris in panel **a’**, which is a magnification of the area within the box in panel **a**. Macrophage-engulfled myelin and axonal debris (panel **c**., arrows) is further evidence of ongoing Wallerian degeneration.

Within the GEL™ treated group, the histology of the nerves was in stark contrast to the shams and control groups. The gross appearance of the tissue at the time of dissection revealed a discrete area of swelling, or a bulge, along the course of the distal tibial nerve, in all specimens of the GEL group only. These discrete structures were about twice the diameter of the nerve just distal and proximal to the outcropping. Longitudinal sections through the portion of the tibial nerve that contained these bulges revealed that the swelling is the result of changes within the endoneurium, including evidence of intraneural edema with increased spacing between the neural fascicles, and axonal edema in the fibers within the bulge region ( Figure 13 a. and b.). In addition, there were numerous profiles in which ongoing axonal fragmentation, a hallmark of Wallerian degeneration, was evident (see arrows in Figure 13 a’ and c.) ^54^.

**Figure 14.**
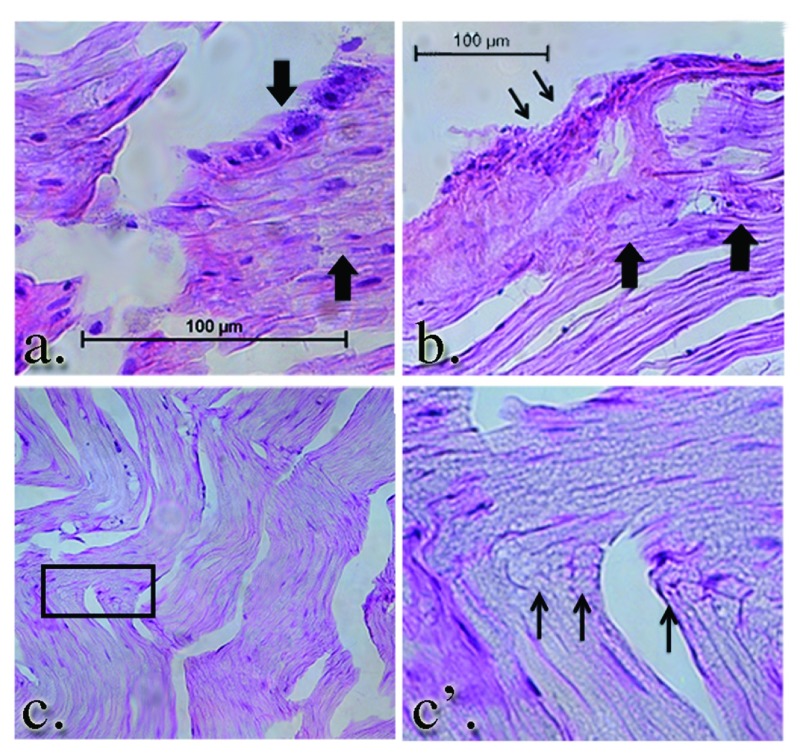
Simultaneous Wallerian degeneration and axonal regeneration is evident in the GEL group nerves. Intraneural (large arrows **a**. and **b**.) and perineural leukocytic infiltration (thin arrows, **b**.) is seen in the GEL group nerves. Notably, there are numerous profiles of regeneration axons in the same nerve (panel **c**. and **c’**) at the same proximal/distal level, suggesting ongoing nerve remodeling in the GEL procedure animals. Panel **c’** is a higher power magnification of the clusters of small, unmyelinated axons within the box in panel **c**.

Consistent with ongoing Wallerian degeneration, we observed numerous macrophages within the endoneurium, which phagocytize myelin and axonal debris ( Figure 14 a. and b. large arrows). We also noted a significant leukocyte accumulation in and around the perineurium ( Figure 14 b. thin arrows).

Findings consistent with any gel residual were not noted in the GEL procedure specimens. In addition to the ongoing Wallerian degeneration observed in the GEL animals, we also noted ongoing axonal regeneration. There were clusters of small, unmyelinated or lightly myelinated fibers growing into the nerves of the GEL cohort only. The grouping of these small fibers is consistent with regeneration, rather than the randomly single-arrayed, unmyelinated fibers that are found in the healthy, homeostatic adult nerve ^55^.

## Discussion

Our conclusions of analgesic data reliability in the GEL™ model are supported by the internal validity of our methodology to eliminate bias with eligibility criteria, randomization, allocation concealment, blinding ^42^, and the repeated drug screenings over time. External validity was addressed with lower doses of drugs to reduce adverse effects, 3 ^rd^ party veterinarian monitoring, use of mature outbred rats and modeling the tissue physiology changes known to occur in neuropathic pain patients with prior tissue injuries ^42^. Other factors influencing external validity were the enhanced ventilation for volatile organic compounds, the high frequency monitoring and having the building isolated from other animals. The limitations of our study’s translational merit are the use of only elicited reflex behaviors as a measurement of neuropathic pain, using only male rodents, individual housing of the rats, and using the same doses of the analgesics studied.

The nonsurgical aged NeuroDigm GEL™ model appears to translate key traits of many humans complaining of persistent neural pain by being of gradual onset, persisting for months, and lacking deformities or antalgic gait. Our mature GEL model’s mechanical hypersensitivity responses to the analgesics studied suggest a negative predictive value for morphine and celecoxib, and a positive predictive value for gabapentin and duloxetine, similar to a meta-analysis of human studies ^56^. With an analgesic profile similar to humans at near human equivalent doses on repeated screenings, our model may have relevance as a translational rodent neuropathic pain model.

Mature adult rats ^44^ were chosen to resemble more closely, human chronic pain patients ^1, 57^. The allodynia response in this aged model was weak, while the response to pinprick with hyperalgesia was robust. Younger (270 g) rats used with the NeuroDigm GEL™ method have had robust allodynia over 2 months ( Supplementary file S5). Neuropathic pain models in older rats have been recognized as having less pronounced mechanical allodynia than younger ones ^58, 59^.

The effect of the GEL neural lesion on the nervous system appears wide spread as central sensitivity or neural plasticity is demonstrated 2–3 weeks after ipsilateral onset by the appearance of elicited pain behavior on the contralateral side in all GEL procedure rats, as well as the five sham rats that developed the late onset pain behavior. The contralateral spread of pain behaviors in this model indicates there are central changes as well. Whether this is the result of anatomic changes or the alterations in neural signaling remains to be determined. Central sensitivity with contralateral pain is known to exist in humans with originally unilateral neural pain ^60– 63^. 

The sham effect with the delayed onset of neuropathic pain in 5 of 8 rats was not anticipated, but evaluation of the data depicted a more delayed tissue response ( S1) than the GEL group. While the GEL™ appeared to be a moderately strong stimulus for tissue repair causing a pain response initially at 23 days ( Figure 2), the data shows that the sham fluid was a weaker stimulus for a tissue response than the gel, with the gradual consistent onset of pain behavior after 60 days in the five out of eight shams with pain ( Figure 2, Figure 6). If this experiment ended prior to 3 months, the sham effect would not have been evident. As in our shams, many human neuropathic pain conditions begin months after an injury ^64^.

An opioid related hypersensitivity ^65, 66^ or resistance is suggested by the data as seen in the GEL and 5/8 sham animals ( Figure 7). Such lack of effective analgesia with morphine over time is characteristic of many neuropathic pain patients ^67– 69^. Since each of the three doses of morphine in this study is separated by weeks, the weakening response to morphine does not reflect tolerance but a resistance. This morphine resistance may correlate to the gradual development of nerve injury ^70^. Our study results also show that repeated screenings of analgesics over time may help in determining translational effectiveness ^71^.

Erythropoietin ^72– 75^, methylprednisolone ^76^, glucocorticoids in general ^77^, and ARA290, an erythropoietin derived tissue repair peptide ^78– 80^, as well as other biologics ^81^, are known to have neuroprotective effects systemically and locally in nerve injury rodent models. The mechanisms causing EPO to reverse pain behavior are not known ^82^. The EPO injection appears to act only at the site of the procedure in this model, as the EPO placement in 2 of the 5 rats in the “at site” group had their injection repeated with improved targeting and effectivity, and the subcutaneous EPO injection had no effect. In our GEL™ model the EPO injection acts similar to a diagnostic and therapeutic peripheral nerve block.

The “ectopic” site and “pain generator” theories for the persistence of neural pain are supported by the targeted local injection of an erythropoietin analog. The unilateral application of erythropoietin appears to have resolved, for at least 6 days (end of study), the bilateral pain behavior of mechanical hyperalgesia (pinprick). The focal neural swelling seen on the distal tibial nerve in the GEL group may act like a mid-axon nociceptive stimulus or ectopic generator, maintaining the pain behavior until treated by the targeted EPO.

The hormonal factor found to influence pain behavior, as described in the Results, echoes the olfactory ‘male observer’ effect of male experimenters reducing acute pain behaviors in rodents, as compared to females ^83^; and suggests that besides their sex, the age and hormonal status of investigators may influence the reproducibility of pain behaviors.

An unanticipated feature suggested in our 5-month study is the evidence for habituation to the von Frey and brush stimuli. In this study, the light touch stimuli (von Frey, brush) have less painful significance than the pinprick, so their responses may be susceptible to habituation ( Figure 3, Figure 4). We have not been able to locate any specific study of habituation to von Frey or brush stimuli. Ipsilateral responses to light touch stimuli in the GEL group appeared to diminish in post procedure periods 4 and 5. By contrast, the sham group’s response tended to remain the same or increase during the same time periods, as some of them developed pain behavior ( Figure 4, Figure 5, and Figure 6). We did not observe any evidence of habituation to the pinprick stimulus in any group.

The nerve specimens used in this study were processed at end of the near 6-month study, when chronic tissue changes dominated, without evidence of acute inflammation. The histological changes seen were restricted to the NeuroDigm GEL™ procedure group and are consistent with ongoing tissue remodeling in the area where the GEL was placed. Specifically, there was evidence of both Wallerian degeneration and axonal regeneration, hallmarks of nerve remodeling. The GEL evoked changes of extraneural matrix tissue can result in nerve compression, resulting in neural remodeling with the delayed onset of pain. Also consistent with ongoing nerve remodeling is the observed inflammation with leukocytic infiltration, seen in both the endoneurial environment and the extraneural space.

Notably, a large number of tightly packed unmyelinated fibers were within the nerves of the GEL procedure animals, consistent with regeneration. The increased number of these fibers and their unusual clustered appearance raises the issues of adequate insulation and the possibility that some of the pain behavior might be due to ephaptic transmission ^84– 86^. Neural regeneration with ephaptic transmission is likely the underlying cause of both the Tinel’s sign ^87^, observed in some patients at sites of neural compression due to entrapment, and also the Pinch Reflex Test, found at sites of regenerating peripheral nerves in experimental rodents ^88, 89^. Such ephaptic sites of neural regeneration may also be related to a flinch jump reflex, mechanically elicitable in many neuropathic pain patients (MRH physician practice 1987–2017).

The histology demonstrates that the neural response to the GEL procedure is restricted to a focal area of neural and axonal edema with neuroinflammation in the tibial nerve. Light microscopy showed that the three shams (from the 5/8 with robust pain behavior) had no visible anatomic changes on histology, similar to the paclitaxel model ^90^. Additional studies are needed in which the effects of the GEL™ induced distal mononeuritis are explored within the brain, spinal cord and the dorsal root ganglion.

Our histology findings are consistent with regenerative neural remodeling found in neural biopsies of humans with persistent pain due to known nerve entrapments ^87, 91, 92^. The soft tissue reaction in our model uses the encoded response that occurs after any tissue injury in all vertebrates ^93, 94^, therefore, many neuropathic pain syndromes may be considered as a soft tissue disease. The last stage of the tissue repair process is fibrosis or remodeling ( S1) ^95^, which may be a unifying etiology in many complex neuropathic pain syndromes.

## Conclusion

Our hypothesis on a perineural tissue matrix etiology for neuropathic pain has been supported. Our objectives to create the delayed onset of neuropathic pain behavior with minimal peripheral nerve trauma using a physiologic hydrogel in a mature rat and to characterize the model’s responses to known analgesics and a targeted neuroprotective were met. The GEL™ model likely creates a neural compression (pinch) ^96^ that induces a focal site of regenerative inflammation, with gradual bilateral behavioral changes; consistent with an occult neural lesion of the somatosensory nervous system as in the definition of neuropathic pain ^97^.

The GEL method supports the 3Rs initiative for refinement in the humane use of animals by minimizing suffering and improving animal welfare ^98^ ( S3). If predictive at determining analgesia, the GEL model may reduce the number of animals needed for studies. The refined NeuroDigm GEL™ model has an accessible neural biomimetic target for translational studies exploring cell signaling, neural imaging, biomarkers, analgesics, detection devices, biologic treatments and alternatives to opiates.

## Data availability

The data referenced by this article are under copyright with the following copyright statement: Copyright: © 2017 Hannaman MR et al.

Data associated with the article are available under the terms of the Creative Commons Zero "No rights reserved" data waiver (CC0 1.0 Public domain dedication). 

### Dataset

**Dataset 1. Raw data of NeuroDigm Model of neuropathic pain in mature rat.** The raw data used for the statistical studies are provided. http://dx.doi.org/10.5256/f1000research.9544.d137472 ^99^

## Figures and Tables

**Supplementary file S1.**
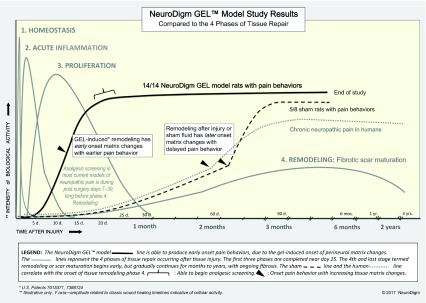
Tissue repair comparison graph. Compares the onset of pain behaviors in this study to the onset of tissue remodeling with fibrosis.

**Supplementary file S3.**
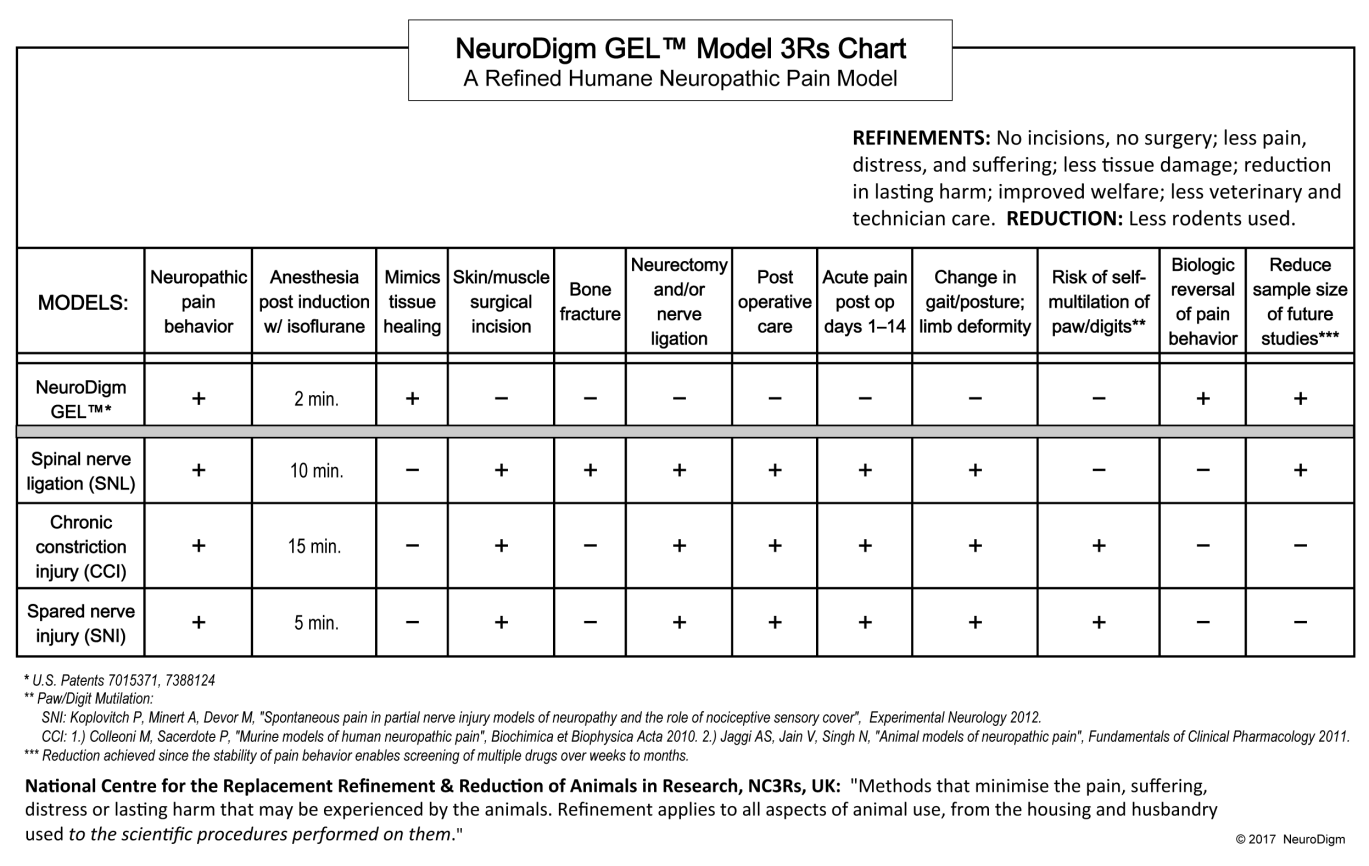
GEL model 3Rs Chart. Chart of NeuroDigm GEL™ model refinements compared to surgical models.

## Supplementary material

**Supplementary file S2: NC3Rs ARRIVE guidelines.**

Checklist for *in vivo* animal experiments.

**Supplementary file S4: Basic Anova statistics on routine tests.**

Detailed Anova statistics on each group on the routine test days without analgesics

**Supplementary file S5: GEL™ model study in young rats.**

Analgesics over months in young NeuroDigm GEL rats with robust allodynia.
