# Supplementary material for: The refined biomimetic NeuroDigm GEL™ model of neuropathic pain in a mature rat
Source: F1000Res. 2017 May 4;5:2516. Originally published 2016 Oct 13. [Version 2] doi: 10.12688/f1000research.9544.2 (PMC5461904; doi:10.12688/f1000research.9544.2)

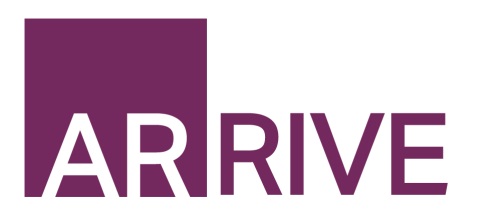


The ARRIVE Guidelines Checklist

Animal Research: Reporting In Vivo Experiments

Carol Kilkenny^1^, William J Browne^2^, Innes C Cuthill^3^, Michael Emerson^4^ and Douglas G Altman^5^

*^1^The National Centre for the Replacement, Refinement and Reduction of Animals in Research, London, UK, ^2^School of Veterinary Science, University of Bristol, Bristol, UK, ^3^School of Biological Sciences, University of Bristol, Bristol, UK, ^4^National Heart and Lung Institute, Imperial College London, UK, ^5^Centre for Statistics in Medicine, University of Oxford, Oxford, UK.*

|  | | ITEM | RECOMMENDATION | Section/ Paragraph |
| --- | --- | --- | --- | --- |
| 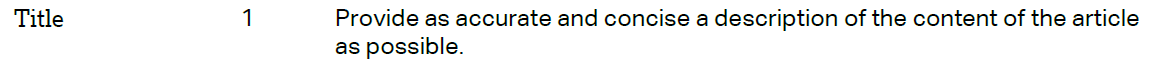 | | | Title |  |
| 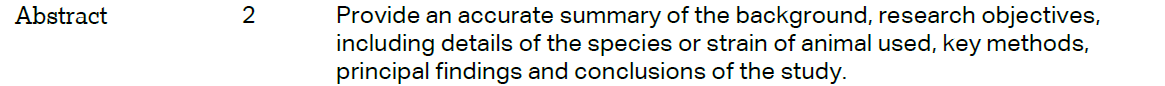 | | | Abstract |  |
| INTRODUCTION | | |  |  |
| 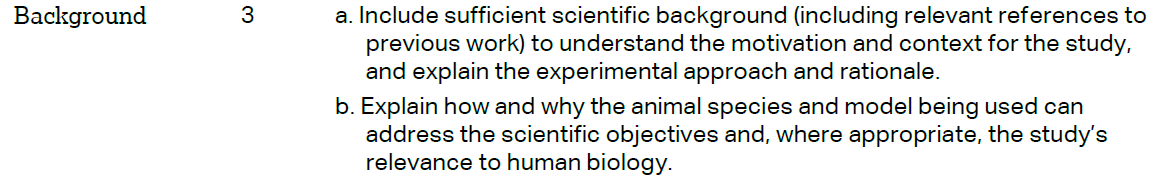 | | | Introduction:  a. Pgraph 1-4  b. Pgraph 5 |  |
| 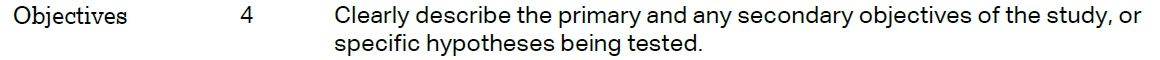 | | | Introduction Paragraph 3; Sample Size |  |
| METHODS | | |  |  |
| 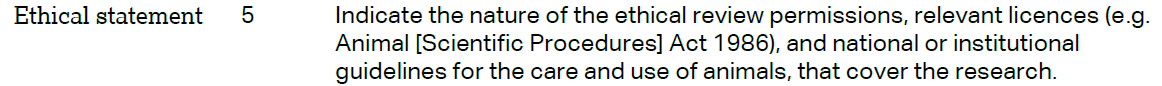 | | | Mat/Methods:  Paragraph 1 |  |
| 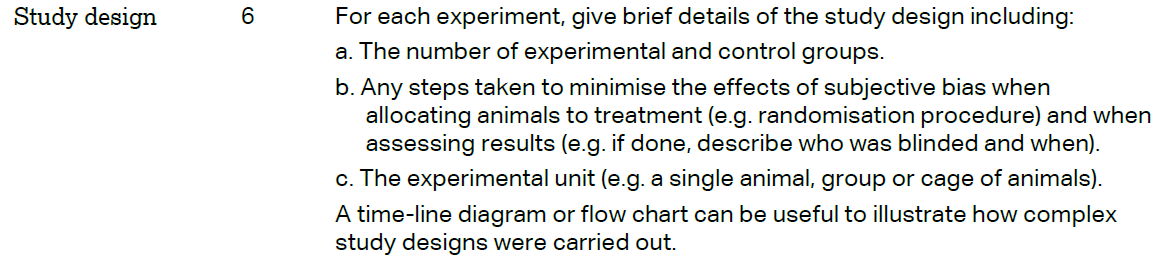 | | | Mat/Methods:  a. Study Design    b.Study Design  c. Timeline Fig 1 |  |
| 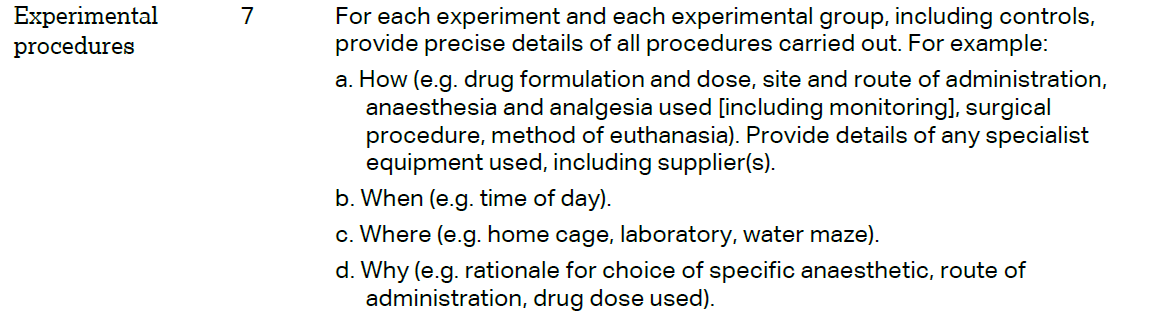 | | | Mat/Methods:  a:  Experimental Procedures:  all sections  b. c:  Housing Sec.  d: Study design |  |
| 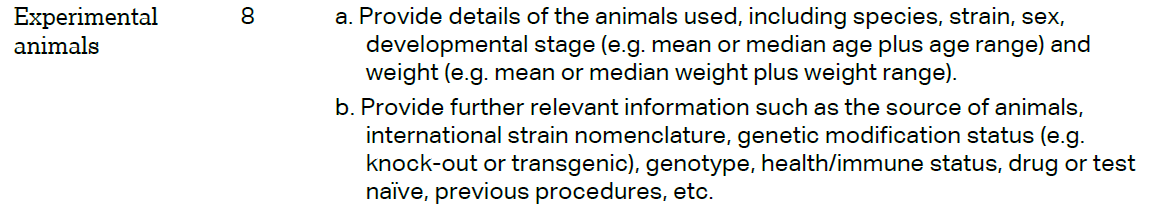 | | | Mat/Methods:  a, b.  Paragraph 2 |  |

The ARRIVE guidelines. Originally published in *PLoS Biology*, June 2010^1^

| 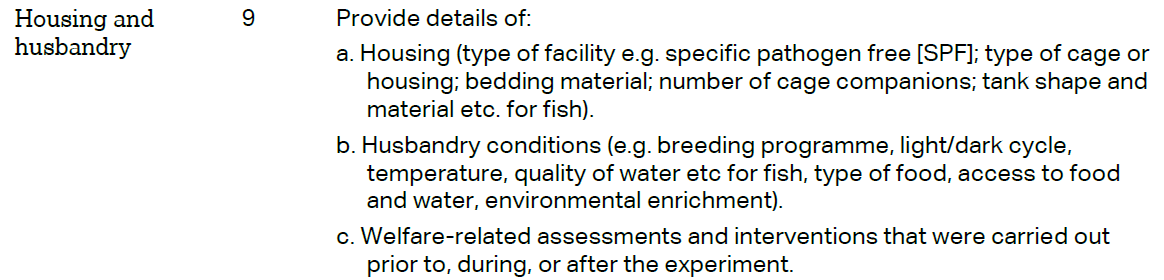 | Mat/Methods:  a,b: Pgraph 3  Housing and Husbandry  c: Study Design | |
| --- | --- | --- |
| 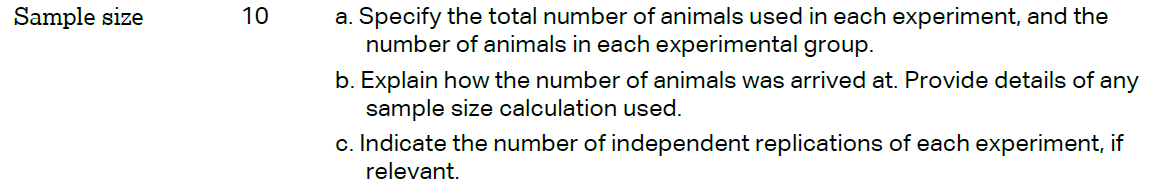 | Mat/Methods  a,b,  Paragrah 7  on Sample Size | |
| 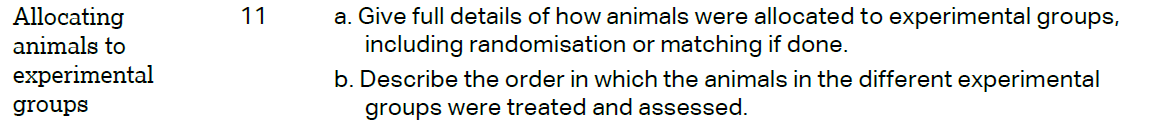 | Mat/Methods:  a.b.Study design  b.Experimental Procedures: Pgraph 2 | |
| 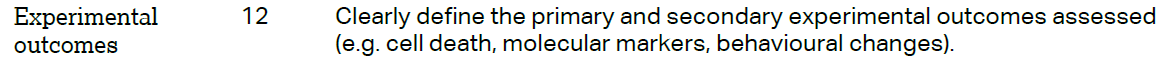 | Mat/Methods:  Sec. on Experimental procedures 1-3 Pgraphs | |
| 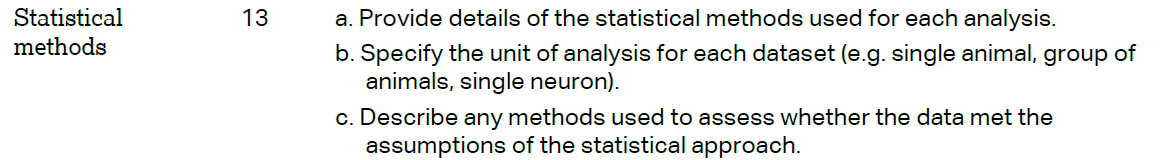 | a,b,c:Mat/Methods:  on Statistical Methods:  Pgraph 1,2,3 | |
| RESULTS |  | |
| 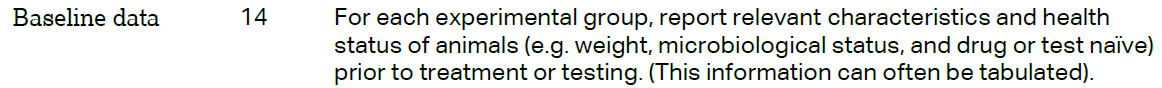 | Results: Behavioral Observationsand in Mat/Methods:  Paragraph 2 | |
| 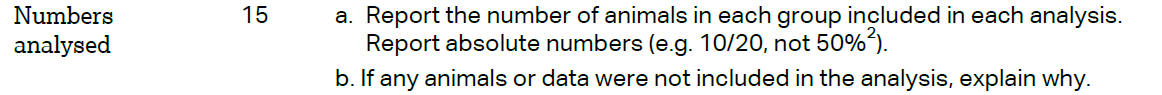 | a:  In Results:  Multiple Pgraphs on  1. Results of Behavioral data  2. Results of Analgesic data  b.:Mat/Methods Paragraph 2 | |
| 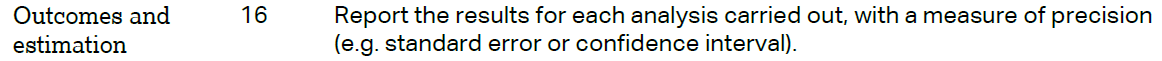 | Results: each Behavior and Analgesic with Calculations  In Multiple statistics | |
| 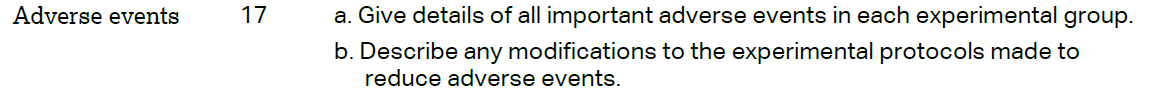 | Mat/Methods: a.,b. Analgesics admin in Paragraph 2 | |
| DISCUSSION |  | |
| 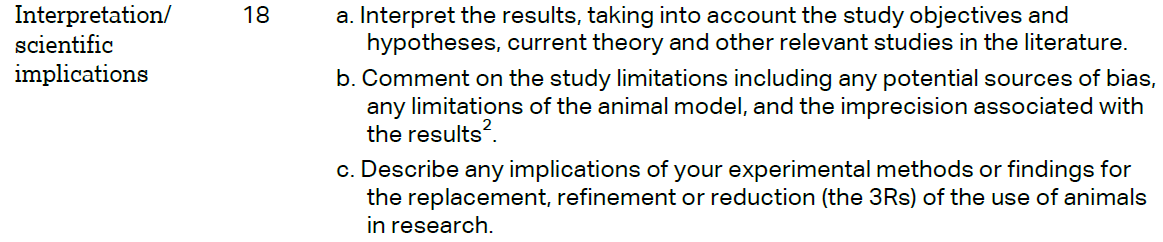 | a. Discussion:  Pgraphs 1-8  b. Discussion Paragraph 1  c. Conclusion: Paragraph 2 | |
| 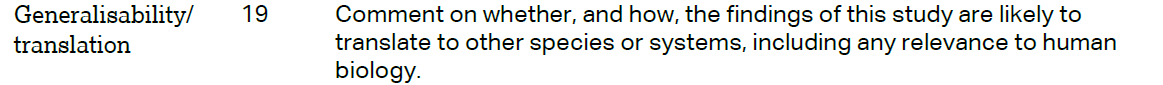 | Discussion: Pgraphs:  1,2,12  Conclusion:  Pgraph 2 | |
| 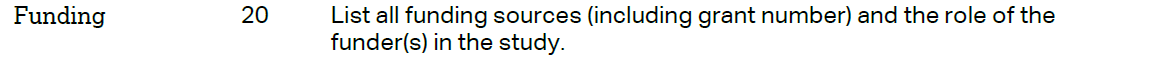 | | In Grant Information: NeuroDigm funded |


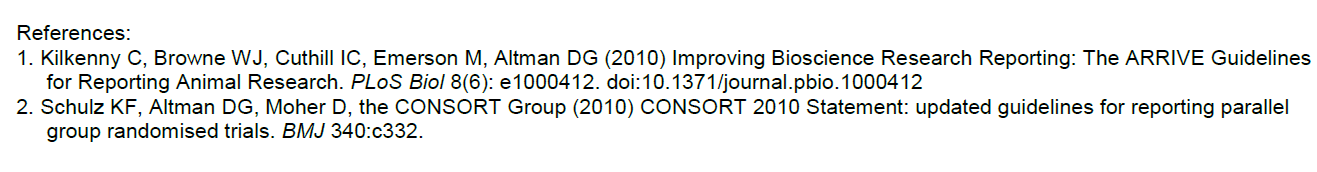

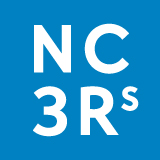

Supplement: Supplementary file 2 [file f1000research-5-12326-s0001.tgz › 565650b5-07a6-4a8b-b5f3-2157ea4c8a9f.docx]
