# Supplementary material for: The refined biomimetic NeuroDigm GEL™ model of neuropathic pain in a mature rat
Source: F1000Res. 2017 May 4;5:2516. Originally published 2016 Oct 13. [Version 2] doi: 10.12688/f1000research.9544.2 (PMC5461904; doi:10.12688/f1000research.9544.2)
Supplement: Supplementary file 3 [file f1000research-5-12326-s0002.tgz › 727c8744-2756-41c5-a6cc-8ae2e7a988e2.docx]

**Supplement S5 : Detailed Anova Stats on Basic Groups (no drugs)**

**Allodynia von Frey Analysis:** Although the planned comparisons were protected by a significant overall *F* value, the large number of means to be compared in this von Frey analysis (3 x 6 x 6 = 108 means) raises concerns about type I errors. For that reason, a more stringent criterion was used for these multiple comparisons (*p* < .01 instead of *p* < .05 to claim a .05 level of significance) and a relatively few pairs of means were compared.

The full ANOVA model consisted of three main effects (Group, Period, and Force-sidedness), three two-factor interactions, and the one three-way interaction. The resulting *p* values for all main and interaction effects were less than .001 including the three-way interaction (*F* (50, 750) = 2.21, *p* < .001). The existence of a significant three-way interaction indicates that all effects in the data cannot be explained with reference to any main or two-way interaction effects, so these were ignored. Planned comparisons that were conducted among the means were limited to those that make the most sense in the context of the experiment. For each group (Control, GEL, Sham), fiber forces (V1, V2, V3) and side (ipsilateral/contralateral) there were 6 periods consisting of a baseline period and 5 post GEL procedure periods. Each of the post procedure periods was compared with the baseline for the same group to determine if the response increased or decreased from the animals' own baseline. GEL procedure effects were determined by comparing the means of the Control and GEL groups, the Sham and GEL groups, and the Control and Sham groups for the same fiber force and side during the same period. Effects of fiber force were determined by comparing means for pairs of fibers on the same side during the same period in the same group. Similarly, effect of side (ipsilateral/contralateral) was determined by comparing the means for the two sides at the same level of fiber force, group, and period. When these comparisons were made during the baseline period or within the Control group, the effects would indicate whether the responses varied according to fiber force without regard for the GEL procedure effects, and the ipsilateral/contralateral comparison would serve as an internal replication of any fiber force effects (there were no experimental differences between ipsilateral and contralateral during the baseline period or in the Control group). All other contrasts among the means were ignored.

**Fiber Force von Frey:** Effects of fiber force were examined first in order to determine whether the animals could discriminate the different levels of force, and if so, to determine the size of this effect without respect to any GEL or Sham procedures. In six comparisons of fiber V3 with fiber V1 during the baseline period (3 groups, 2 sides), all means showed an absolute increase from fiber V1 to fiber V3 and the effect was significant in five of the six comparisons. The size of the effect of fiber force was determined by calculating the means of all 33 animals in the study for each fiber force and side. On the ipsilateral side, the means and standard deviations of paw withdrawals for fibers V1, V2, and V3, respectively, were 0.10±0.28, 0.43±0.40, and 0.56±0.45. On the contralateral side, the corresponding data were 0.11±0.17, 0.46±0.36, and 0.54±0.46. The difference between the means of fiber V3 and fiber V1 is about 0.45 paw withdrawals, which is about equal to the largest standard deviation observed in the data.

**Comparison of GEL Procedure to Baseline and to Control Group for von Frey:** Post-procedure data from each of the five monthly periods were compared with the baseline period in all three groups. In addition, the means from the GEL and Sham groups were compared with the mean of the Control group at the same time after the procedures in order to control for effects of time and repeated stimulation. Stimulation in the Control group during the five monthly "post-procedure" periods never significantly exceeded the baseline value at any fiber force or on either the ipsilateral or contralateral side.

Stimulation in the GEL group produced significant increases from the baseline value in 10 of 15 means when the stimulus was applied to the ipsilateral side. Post-procedure increases in the GEL group were also significant when compared with the Control group in 11 of 15 possible comparisons. The single GEL-group mean that was significantly different from the Control group but not different from baseline was the mean for Period 5 for fiber V2 on the ipsilateral side. Significance was maintained between groups because the Control group was lower in that period than in any other period. The peak between-group effect size with fiber V3 during post-procedure period 3 for the GEL group was 1.49 paw withdrawals (means and standard deviations for Control, 0.64±0.23 and for GEL, 2.13±1.02) for a standardized effect size of 1.91 pooled standard deviations.

Stimulation to the contralateral side in the GEL group produced significant increases from the baseline value and from the Control group in only 3 means (means marked with asterisks in Fig 3).

**Allodynia: Brush Analysis**

Analysis of the paw withdrawal responses to the brush stimulation employed a mixed-model ANOVA similar to the analysis for the von Frey fibers except that there were not three levels of brush as there were three levels of fibers. The between groups factor was the three groups, Control, GEL, and Sham. The repeated measures factors were the six 30-day periods and the two sides on which the stimulation was applied (ipsilateral or contralateral). The three-way interaction of Groups, Periods, and Sides was statistically significant (*F* (10, 150) = 1.943, *p* = .044). In the Control group, the means for the five monthly "post procedure" periods never significantly exceeded baseline on either side. The only mean on the ipsilateral side that was significantly different in the between-group comparison that was not significantly different from the GEL group baseline was the GEL group mean for Period 5.

The maximum between-group effect size at the peak of the effect was 0.48 paw withdrawals on period 3 (means and standard deviations, Control group 1.34±0.61, GEL group 1.82±0.86) indicating that the maximum standardized effect size was at least 0.56 standard deviations. For the contralateral paw, no increases were observed, and the mean value actually decreased from baseline in the GEL group during periods 2, 4, and 5 (not marked in Fig 4). The mean of the Sham group exceeded that of the Control group only in period 5 on the ipsilateral side.

**Hyperalgesia: Pinprick analysis**
The pinprick analysis was identical to that for the brush, with the 3 groups as a between subjects factor and the periods and ipsilateral/contralateral sides as repeated measures factors. The data for pinprick on the ipsilateral and contralateral sides are presented in Fig 5. All of the main and interaction effects in the model were significant at *p* < .001 except for the interaction of Groups x Sides, which was significant with a *p* = .004. Because the three-way interaction was significant (*F* (10, 150) = 4.592, *p* < .001) all other effects were ignored. Planned comparisons revealed that the Control group never deviated from its own baseline value in any post procedure period on either side. By contrast, the GEL group on the ipsilateral side was significantly greater than baseline during all 5 post-procedure periods, and the GEL group on the contralateral side was significantly greater than baseline during periods 2 through 5.

Between-group comparisons for pinprick indicated that the three groups were not significantly different during the baseline period on either the contralateral or ipsilateral side. On the ipsilateral side during the post-procedure periods, the GEL group significantly exceeded the means of the Control groups during all 5 periods. On the contralateral side, the GEL group significantly exceeded the means of the Control group during periods 2 through 5.
