# Supplementary material for: The refined biomimetic NeuroDigm GEL™ model of neuropathic pain in a mature rat
Source: F1000Res. 2017 May 4;5:2516. Originally published 2016 Oct 13. [Version 2] doi: 10.12688/f1000research.9544.2 (PMC5461904; doi:10.12688/f1000research.9544.2)
Supplement: Supplementary file 4 [file f1000research-5-12326-s0003.tgz › d36025e2-cf2e-4dd6-a74a-422f4fda7171.docx]

**Study with younger NeuroDigm GEL™ model:**

Young 270g Male Rat NeuroDigm GEL^TM^ Validation Study by independent CRO in 2013

• Screened with Chaplan up/down method

for mechanical allodynia threshold

• Study was over 67 days

• Drugs screened twice about 30 days days apart:

Morphine, Celecoxib, Gabapentin and Duloxetine

Results demonstrate how time can effect a drug’s analgesic response

[NeuroDigm GEL^TM^ model Validation Video](http://youtu.be/Qb5DFdVk9wY)

<http://youtu.be/Qb5DFdVk9wY>

Investigators:

Yan Chen PhD – designed blinded study and analyzed data,

Cheng Yang – male, performed blinded analgesic screenings

Mary Hannaman MD – performed initial procedures with Dr. Yan Chen assisting.

Note: This younger NeuroDigm GEL™Model had robust and prolonged mechanical allodynia. While the aged Neurodigm GEL Model in the rat in this paper had very weak allodynic responses.
