# Supplementary material for: The refined biomimetic NeuroDigm GEL™ model of neuropathic pain in a mature rat
Source: F1000Res. 2017 May 4;5:2516. Originally published 2016 Oct 13. [Version 2] doi: 10.12688/f1000research.9544.2 (PMC5461904; doi:10.12688/f1000research.9544.2)
Supplement: Peer review history

# Peer review: Referee response for version 2

**DOI:** 10.5256/f1000research.12326.r23215

**Published:** 2017-06-05

**Authors:** Gillian Currie

**Affiliations:** 1 Centre for Clinical Brain Sciences, University of Edinburgh, Edinburgh, UK

The revised version of the manuscript is improved and the authors have responded to my comments or made appropriate revisions.

I have read this submission. I believe that I have an appropriate level of expertise to confirm that it is of an acceptable scientific standard.

# Peer review: Referee response for version 2

**DOI:** 10.5256/f1000research.12326.r23166

**Published:** 2017-06-01

**Authors:** Odd-Geir Berge

**Affiliations:** 1 OBGConsulting AB , Rönninge, Sweden

This second version of the manuscript is better structured and thus easier to read. Reservations detailed in my original comments with regard to references, terminology and generalizations have not been addressed to satisfaction.

I have read this submission. I believe that I have an appropriate level of expertise to confirm that it is of an acceptable scientific standard, however I have significant reservations, as outlined above.

# Peer review: Referee response for version 2

**DOI:** 10.5256/f1000research.12326.r22519

**Published:** 2017-05-30

**Authors:** Michael Brines

**Affiliations:** 1 Araim Pharmaceuticals Inc.,, Tarrytown, NY, USA

The authors have satisfactorily addressed my concerns and suggestions using a combination of article revision and replies to specific issues raised by the reviewers. I have no further comments.

I have read this submission. I believe that I have an appropriate level of expertise to confirm that it is of an acceptable scientific standard.

# Peer review: Referee response for version 1

**DOI:** 10.5256/f1000research.10281.r16966

**Published:** 2016-11-15

**Authors:** Gillian Currie

**Affiliations:** 1 Centre for Clinical Brain Sciences, University of Edinburgh, Edinburgh, UK

The research article “The refined biomimetic NeuroDigm GEL ^TM^ Model of neuropathic pain in the mature rat” describes a new neuropathic pain animal model designed to be more relevant to the development of chronic neuropathic pain in humans. The authors describe a model using mature adult rats with a percutaneous implant of GEL ^TM ^into the tibial nerve. The model is characterised for 5 months by assessing pain-related behavioural responses to mechanical stimuli and the effect of morphine, celecoxib, gabapentin and duloxetine. Histology of the nerve was also assessed. A pilot study is also reported assessing the pain-related behavioural response to the injection of human erythropoietin.

 The rationale for the development of a model of neuropathic pain that more closely mimics the human condition is sound. I agree that the most commonly used neuropathic pain models do not mirror the pathophysiology of the delayed onset of neural pain without debility as seen in many neuropathic pain patients. The authors provide detailed description of their methods and rationale and include an ARRIVE guidelines checklist.

 I am concerned that the Sham animals are not used as controls to characterise this model. The authors acknowledge that the sham animals are not homogenous in their pain-related behavioural responses (5 out of 8 animals develop pinprick-induced pain-related behaviours). However, I believe that this is the most appropriate control for this new model of neuropathic pain.

 I disagree with the claim that this model meets the NC3Rs criteria for refinement. Refinement refers to methods that minimise the pain, suffering, distress or lasting harm that may be experienced by animals. The longer duration of this model and the development of similar pain-related behaviours as observed in other neuropathic pain models does not meet refinement criteria.

 Specific recommendations:

**Title**

 Description of the model as refined needs to be clarified in the article. How is this a refined model?

**Abstract**

 Research objectives should be stated in the abstract.

 Strain of animal should be stated in the abstract.

 I do not think the results of the pilot study should be stated in the conclusions of the abstract. The main study characterising the model, which is appropriately powered, should be the focus.

**Introduction and background**

 It would be useful to use more specific references for the type of pain that this model is meant to be modelling.

**Materials and Methods**

 Animals were randomly assigned to groups as stated in the Methods section Study Design Paragraph. Please state how animals were randomly allocated to group.

 Also, state whether animals were randomly assigned to analgesic treatment groups and how this was carried out.

**Results**

 The data supports the claim that the model does develop pain-related behaviours that develop gradually and persist for months compared to control animals. However, should the comparison be to sham animals?

 Do the analgesic responses reflect human responses?

 All figures: For presentation of results in the figures I recommend the used of standard deviations not standard error of the mean ^1^.

 Figure 5: The Sham animals also show an increase in pain behaviour from baseline and this should be indicated with asterisks.

 Figure 7-10: How many animals were tested in the analgesic drug experiments? This should be clearly stated in the results section and in the figure legend. Were the same animals used for each drug? If so, this should be clearly stated.

 The authors give a thorough and transparent description of their data and analysis choices. However, I recommend that a statistician assesses the statistical methods. For example, I question the use of the Fisher’s Protected Least Significant Difference test as this does not account for multiple comparisons. I also query the use of the Bonferroni-protected contrast because, as I understand it, this should only be used following a significant ANOVA result.

**Discussion**

 Study limitations should be explored. For example, the use of only reflex behaviours to measure pain-related behaviours.

 The paragraph outlining the implications to the 3Rs should be changed as I do not believe that this model is a refinement of the use of animals in research. This paragraph should also be moved from the conclusions section. Although, it should be noted that in the future if it does provide a more reliable model of human neuropathic pain then it has the potential to reduce the number of animals used in models that are not clinically relevant.

I have read this submission. I believe that I have an appropriate level of expertise to confirm that it is of an acceptable scientific standard, however I have significant reservations, as outlined above.

## References

[1] LangTAltmanD: Basic statistical reporting for articles published in clinical medical journals: the SAMPL Guidelines. *Science Editors' Handbook, European Association of Science Editors*.2013;

# Response

**Published:** 2017-04-12

**Authors:** Mary Hannaman

**Affiliations:** NeuroDigm, USA

Your perspective is appreciated and your concerns have been addressed. EPO pilot study power statistics have been added to the revision. The initial sample size was based on prior investigations.

The Supplemental S3 refinement chart we added depicts how the refinement of a scientific procedure (as referred to by NC3Rs) that limits tissue damage can reduce acute pain, eliminate paw dragging, limb deformities, and self-mutilation. If rodent refinements reduced or eliminated pain their potential as models could be lost.

Your pertinent question about the type of pain the model represents is best answered simply. We strived to elicit the types of evoked pain behaviors referred to as allodynia and hyperalgesia (1) that may develop gradually in patients after soft tissue trauma.

We were trying to mimic in this study the chronic pain sustained for years as seen in humans. Most investigations would not need to be months long and studies can be shortened to any time after post procedure day 23. However, the longer possible duration of this study can possibly reduce the number of animals used in future studies.

There is no homogeneous “sham group” represented by the data that could be used as a control over time. After 3 months 5/8 shams developed pain behavior and there was no single sham animal that had intermediate behavior in the values represented by the graphs. We took the unusual step of presenting the sham data individually to be perfectly clear about what happened in that interesting group. We purposely did not include asterisks in Figure 5, because it would be misleading. The shams’ individual data in Figure 6 resembles the human response — not all humans get neuropathic pain after a soft tissue injury.

The EPO pilot study also helps characterize the GEL model by showing the analgesic effect of a localized biologic, which has not been demonstrated in the current models.

The second author (DAF) consulted on experimental design and data display and conducted the statistical analysis of the behavioral data. Aside from expertise in neurobiology and IACUC regulations, he has taught undergraduate statistics and published several articles (2— 6) on experimental design, simulation, and ANOVA.

Regarding the standard deviation instead of standard error, this recommendation by Lang and Altman (7) is curious because they offer no rationale for it in their paper. Use of standard deviations allows estimation of standardized effect sizes, whereas use of standard errors allows estimation of inference (null hypothesis test or confidence interval). Those authors might have had in mind that the use of standard errors without indication of corresponding sample sizes would rule out the estimation of standardized effect sizes by subsequent readers or meta-analyses. That is not the case in our paper because we explicitly include estimates of standardized effect sizes in Table 1. Thus, there is no reason to prefer standard deviations over standard errors, and the latter assist in informal estimation of significance for the many contrasts that we did not explicitly test.

Fisher's protected least significant differences (PLSD) and Bonferroni: Note that planned comparisons can always be tested, unlike post-hoc comparisons (data snooping). We tested only planned comparisons in this paper. We quote the recommendations of experts Milliken and Johnson (8):

 

1. Conduct an F-test for equal means.
2. If the F-statistic is significant at the 5% level, make any planned comparisons you wish to make by using the LSD method. This includes not only comparisons between pairs of means but also comparisons based on any selected contrasts of the µ _i_’s. If one has equal sample sizes, the Waller-Duncan method can also be used. For data snooping and unplanned comparisons, use Scheffe’s method.
3. If the F-statistic for equal means is not significant, the experimenter should still consider any individual comparisons that he or she had planned, but should do so using either the multivariate t-distribution method or Bonferroni’s method. The experimenter should not do any data snooping in this case. Since the F-test for equal means is nonsignificant, Scheffe’s procedure would not yield any significant differences anyway.

 

Your insightful question “Do the analgesic responses reflect human responses?” highlights a crucial issue that cannot be accurately answered despite extensive discussion in the literature. Presently “pain” in patients and experimental rodents is assessed by different assays. With similar “pain assays” the validity of such translational comparisons can be improved.

**References**

1. Baron R. Mechanisms of disease: neuropathic pain—a clinical perspective. Nature clinical practice Neurology. 2006 Feb 1;2(2):95-106.

2. Fitts DA. Misuse of ANOVA with cumulative intakes. Appetite. 2006 Jan 31;46(1):100-2.

3. Fitts DA. Improved stopping rules for the design of efficient small-sample experiments in biomedical and biobehavioral research. Behavior research methods. 2010 Feb 1;42(1):3-22.

4. Fitts DA. The variable-criteria sequential stopping rule: generality to unequal sample sizes, unequal variances, or to large ANOVAs. Behavior research methods. 2010 Nov 1;42(4):918-29.

5. Fitts DA. Ethics and animal numbers: informal analyses, uncertain sample sizes, inefficient replications, and type I errors. Journal of the American Association for Laboratory Animal Science. 2011 Jul 15;50(4):445-53.

6. Fitts DA. Minimizing animal numbers: the variable-criteria sequential stopping rule. Comparative medicine. 2011 Jun 15;61(3):206-18.

7. Lang T, Altman D: Basic statistical reporting for articles published in clinical medical journals: the SAMPL Guidelines. *Science Editors' Handbook, European Association of Science Editors*. 2013.

8. Milliken GA, Johnson DE. Analysis of messy data, Volume I: Designed experiments. Wadsworth. Inc. Belmont, California. 1984.

# Peer review: Referee response for version 1

**DOI:** 10.5256/f1000research.10281.r17230

**Published:** 2016-11-14

**Authors:** Michael Brines

**Affiliations:** 1 Araim Pharmaceuticals Inc.,, Tarrytown, NY, USA

This manuscript describes an interesting rodent model of chronic pain. The basic premise is that local injection of a mixture of biological materials typical of the extracellular matrix following tissue injury activates tissue repair processes which ultimately causes a constrictive nerve injury associated with the development of pain behaviors. However, the view that traumatic injury is not involved in the development of this model must be incorrect, as the sham animals also develop pain behaviors, albeit to a lesser degree and with a delay compared to the active procedure arm. The biological processes involved are well schematized in Figure S1 and underscores that the involvement of both injury and repair processes in the development of the neuropathic state.  Unfortunately, pathological evaluation was performed only months after the initiating lesion, long after the acute and subacute effects of the procedure had resolved. If the assumption that purely repair processes are involved in this model, it would be necessary to evaluate in a longitudinal manner molecular and other markers of tissue damage and repair. For example, the presence or absence of inflammatory cells and quantification of pro-inflammatory cytokines at the site of injection of the sciatic nerve in the days and weeks following the experimental procedure would determine to what extent inflammation is involved. A better description of the pathophysiology of this model would be that of a delayed neuropathic state developing after mild peripheral nerve trauma.

 In terms of general issues, the behavioral methodological details are unusually detailed and to aid readability of the manuscript, much could be moved to Supplemental Materials. The authors are to be congratulated in following very rigorous blinding procedures for the behavioral testing which often are lacking in the published descriptions of neuropathy models. These strengthen greatly the behavioral observations. A particularly interesting observation was the effect of estrogen-containing cream present on the experimenter on the pain behavior observed and further underscores the need for great care in limiting potentially confounding variables which reduce the fidelity of experimental observations.

 In spite of the detailed description of the experimental procedures, one question arises: do the authors believe that the injection site targets only the tibial nerve? If so, the exact testing location of the plantar surface will be important, i.e., whether in the tibial or sural nerve distribution. Many peripheral nerve models are characterized by sprouting from the adjacent sural distribution and this possibility needs to be evaluated.

 Additionally, there is a redundant exposition of experimental results: as just 2 examples, Figures 2 and 5 present the same data (Figure 2 is perhaps more useful for the reader) as do Figures 5-6. Streamlining the text and exposition of data, i.e. methods and results, would make the manuscript more readable. The “Summary of pain behavior” and “Conclusion” are redundant in my view.

 The conclusion in the discussion that this model is one of focal peripheral nerve injury is premature, as evaluation of the spinal cord and dorsal root ganglia was not undertaken. The presence of contralateral behavioral findings, even within the sham group, implies that there is at the least critical spinal cord involvement in this pain model. The authors correctly point out that additional studies focusing on the central nervous system are needed to more completely define the pathophysiology of this model.

 The inclusion of EPO-mediated pain improvement raises more questions than it answers and appears almost as an afterthought. Unfortunately, there is not enough detail, especially at the pathological level, to confirm the effect is via “activation of repair”. The extremely fast response, occurring over a few days evidently, is not consistent with true repair which requires a much longer timespan. Additionally, the differentiation between local and systemic effects of EPO assumed in this study may not be correct. The dose of EPO administered (400IU/kg) is at the lower limit needed to exhibit beneficial effects in other neurological models and further, administration via the SC route would reduce the peak plasma EPO concentration. An intraperitoneal or intravenous route would have been preferable. Perhaps this observation would be better moved to supplementary material and speculation on the underlying biology limited.

 In conclusion, this model is interesting and the very detailed methodology makes it a useful addition to the study of pharmacological treatment of pain behavior. The clear distinction between classes of agents used as treatment for neuropathic symptom increase optimism that the model can be useful for screening potential efficacy of new compounds or evaluating novel methods of treatment using existing pharmacotherapy.

I have read this submission. I believe that I have an appropriate level of expertise to confirm that it is of an acceptable scientific standard, however I have significant reservations, as outlined above.

# Response

**Published:** 2017-04-12

**Authors:** Mary Hannaman

**Affiliations:** NeuroDigm, USA

The value of your knowledge of tissue repair is useful in understanding the hypothesis of our model. We realize our focus on the later stages of our model presents frustrations. For this study we merely wanted to develop and then treat the chronic pain behavior as seen in patients. Once established as being representative of patients, earlier studies can be done. Also the extensive testing limited the number of rodents able to be used for specimens in this lengthy study.

The paper’s main tissue focus is on the neural histology findings long after pain behaviors are established, limiting any explanation of the exact pathophysiology. The hydrogel does not directly cause traumatic injury or acute inflammation in the first 14 days, as evidenced by the lack of significant pain behaviors, erythema, cyanosis, edema, or altered gait. Interestingly, the shams that developed late onset pain behaviors had no evidence of tissue damage on light microscopy, and may represent an obscure neural “dysfunction”.

Regarding the redundant exposition of experimental results: We agree that Figure 2 is in many ways more informative, and we used Figure 5 to provide a formal statistical analysis of the null hypothesis. Omitting Figure 5 would omit the statistical analysis.

We agree that EPO does not likely alleviate the pain through tissue repair. The erythropoietin’s neuroprotective mechanisms are not known for this neural model and need further study.

The EPO dose in mg/kg units is close to 300 units/kg. The systemic EPO dose was subcutaneous to limit stress, tissue damage and complications. The repeat injection of EPO in 2/5 GEL rats discussed in paper suggests localized EPO placement is critical for this low dose to cause a reversal effect.

You are correct in stating that this model of focal neuritis creating neuropathic pain may involve more than the discrete site on the nerve. The spinal cord and brain are likely involved, and we saved the brains, as well as non-neural tissue, of the rats for further study.

Your other concerns have been incorporated in the revision.

# Peer review: Referee response for version 1

**DOI:** 10.5256/f1000research.10281.r16989

**Published:** 2016-10-26

**Authors:** Odd-Geir Berge

**Affiliations:** 1 OBGConsulting AB , Rönninge, Sweden

This article presents a novel model of neuropathy with slow onset of symptoms (increased responsiveness to mechanical stimuli) that, once established, persist throughout the study period of several months. Another feature of the model is the use of adult rats. The model is characterized by means of some commonly used drugs, histology, and, as a pilot study added after conclusion of the main study, administration of erythropoietin to a subgroup of animals.

 While the experimental procedures as such appear to be technically solid, reservations may be raised concerning terminology, presentation of background including literature references, translational context, discussion, and validity of conclusions. The structure of the paper can be improved by prioritizing and moving content to relevant sections, which would facilitate reading and increase the focus on important findings. In addition, the erythropoietin “pilot study” was carried out under less stringent conditions and may perhaps be reported as a separate short or preliminary paper.

 Major concerns:

 The authors equate responses evoked by mild mechanical stimuli (von Frey stimulation, gentle brushing, pin prick) with pain behavior and clinical pain. Even terms like allodynia and hyperalgesia are used to describe these responses although the paper provides no evidence that there is any pain involved in the model or that these responses are valid surrogate markers for clinically relevant pain. These types of stimuli are undoubtedly useful both in psychophysical studies and quantitative sensory testing but rarely used to evaluate analgesic efficacy in clinical trials. I would suggest using neutral, descriptive terms throughout the manuscript and bring up the translational relevance in the Discussion, taking into account the extensive literature on this topic.

 The pharmacology is interpreted in terms of analgesia, which is questionable since there is no evidence for pain in the model. The drugs used, perhaps with the exception of celecoxib in low doses, have known non-analgesic effects that may interfere with evoked responses. While pharmacological characterization of a model is useful for various reasons, clinical relevance is a different matter and clinical efficacy is generally poorly predicted by animal models of neuropathy. This is arguably the case here where the efficacy of the tested drugs are much more robust than the case is in well-controlled clinical trials and the order of efficacy, in spite of what is claimed, is at variance with the clinical data (for a recent, comprehensive review, see Finnerup et al ^1^). It should be pointed out that comparing drug efficacy in single-dose experiments has limited utility, especially when pharmacokinetic data are lacking. Relating drug doses in this animal study to human clinical data is of course relevant but should be done in the Discussion with due reference to the many limitations of the current approach.

 The use of literature references is in part inadequate. For instance, the short first paragraph of the introduction contains no less than 30 references and it is hard to identify the ones that may have relevance for the statements in the text. Many of these references deal with conditions that are connected to the topic of the present study only in a general sense and it is not obvious how they may support the arguments. Since delayed development of sensory changes seems to be a main feature of the described model, specific reference to one or two key clinical papers would be helpful. It may be necessary to carefully match the text of the manuscript with the references and perhaps delete sections or statements that are not supported by literature; alternatively to reorganize the text so that speculations and personal opinions are clearly differentiated from information supported by evidence in the literature.

 Specific suggestions & comments

- Title (and in other places):
  The described method is referred to as “refined” but compared to what is not defined in the manuscript.
- Abstract:
  Background: The paragraph should be refocused to state the objectives / hypotheses of the study.
  Methods: The first sentence belongs to the Results paragraph. Mention test methods and readouts. Some details e.g. age of rats, doses, routs of administration of drugs, would give the reader a chance of understanding what to expect from the paper. Generic drug names should not be capitalized (correct in the following section).
  Results: As in the rest of the manuscript, avoid controversial terms like “pain behavior” and “analgesia”.
  Conclusion: The text is inaccurate in suggesting that there is direct evidence for pain behavior in the model and that this behavior is related to remodeling; the difference between the sham and gel treatments is mostly quantitative allowing for other interpretations. It is not obvious that the effects of analgesics in the model reflect clinical efficacy.
- Introduction:
  In addition to the more general points raised above, concentrating the text on the present model into a single paragraph with a clear statement of objective may facilitate reading and sharpen focus. The text regarding relevance of animal models do not adequately address the rather extensive discussion that has been ongoing at meetings and in the literature for quite a while and the argument put forward is unspecific. There is room for a more complete treatment in the Discussion where a number of issues relevant to the present study could be addressed, e.g. interpretation of readouts and the importance of pharmacokinetic factors. In the introduction, perhaps pointing to the expected advantages of the present paradigm compared to previous work would suffice.
- Materials and methods:
  Bodyweight of animals also at the time of testing would be interesting.
  P5, left column, 4 ^th^ paragraph: How was the forces of the von Frey filaments confirmed? Are these filaments stable under changing environmental conditions?
  P5, last paragraph: the meaning of the phrase “all with no change in pain behavior noted" is unclear.
  Some text could be deleted or moved to the discussion to provide easier reading, e.g. p5, left column, 3 ^rd^ paragraph ("Measures of …"), right column 2 ^nd^ paragraph ("The original doses ...")
- Results:
  In general, this part of the manuscript would benefit from limiting the text to what is necessary to understand the data, leaving discussion to the Discussion. Even figure legends should be edited to remove interpretations, speculations and explanation of what can be easily seen from the graphs. All symbols should be explained in the legends. Number of animals, indication of statistical analysis (with details in methods and results), and how the means are calculated could be given in the legends and thus allow for a more succinct main text.
  P7 & 8: “Results of behavioral data” - the first four paragraphs should be deleted or condensed and moved to the discussion (with appropriate succinct information added to the figure legends).
  The following paragraphs “Days of data..." could be condensed to a few descriptive sentences and the justification for various procedures moved to the discussion.
  I would suggest that Table 1 and associated text be moved to Supplemental material or deleted altogether; minor changes in the results may change the numbers significantly so the value of these calculations for future studies would be too limited to warrant an extensive presentation as in the present manuscript.
  P9, Section on von Frey: For consistency: mention the results of the GEL group. The sentence “Asterisks … “ should be moved to the figure legend (this also applies to the following sections).
  P10: “Individual data...”: It is difficult to see what this analysis contributes to the paper – in my view it distracts from the main findings and should be deleted or moved to Supplemental material.
  The following paragraph “Summary ...” is redundant; the “Factor influencing ...” is anecdotal and probably best deleted, alternatively mentioned in the Discussion.
  “Results of experiments...”: the information in the first paragraph fits better in other parts of the manuscript.
  Section on morphine: the first sentence is redundant / irrelevant.
  Later in the paragraph (p 11), the explanation of asterisks can be removed twice, it's adequately explained in the figure legend (same applies to text later in the Results).
  The last paragraph on morphine does not appear to ad value.
  p14: “Summary...” is redundant.
- Discussion:
  I would recommend an extensive revision of the Discussion, addressing methodological aspects and taking into account the limitations of the approach, some of which are delineated above. Careful scrutiny of the references to make sure that they are fair and representative as well as addressing the statements in the text would be another recommendation, also pertaining to the rest of the manuscript. As indicated above, there may be material in previous sections of the manuscript that would fit in the Discussion.
- Conclusion:
  Appears more as a continuation of the discussion and should be revised, preferably to reflect the objectives of the study and the initial hypotheses.

I have read this submission. I believe that I have an appropriate level of expertise to confirm that it is of an acceptable scientific standard, however I have significant reservations, as outlined above.

## References

[1] FinnerupNBAttalNHaroutounianSMcNicolEBaronRDworkinRHGilronIHaanpääMHanssonPJensenTSKamermanPRLundKMooreARajaSNRiceASRowbothamMSenaESiddallPSmithBHWallaceM: Pharmacotherapy for neuropathic pain in adults: a systematic review and meta-analysis. *Lancet Neurol*.2015;14(2) : 10.1016/S1474-4422(14)70251-0 162-73 10.1016/S1474-4422(14)70251-0 25575710PMC4493167

# Response

**Published:** 2017-04-12

**Authors:** Mary Hannaman

**Affiliations:** NeuroDigm, USA

The in-depth analysis you provided was carefully examined and applied. Some areas were retained based on the comments of another, as in Table 1, which has the standard deviations desired by some. In this lengthy research report, discussions of translational context and pharmacokinetics are not covered.

The references have been abridged for relevancy, as suggested. The hypothesis and objective have been included in the Abstract and Conclusion. Paragraphs have been moved to more relevant headings. Summaries were deleted. Clarifications regarding refinement are discussed in the revision (Supplement S3).

Early recognition of a hormonal influence on behavioral inconsistencies was crucial to the study’s completion. If the role of estrogen had not been realized the study would have been terminated.

The very thorough paper by Finnerup et al 2015 “Pharmacotherapy for neuropathic pain in adults: a systematic review and meta-analysis” (1) classifies gabapentin and duloxetine as first-line drugs for neuropathic pain with a “high quality of evidence” for both, and strong opioids as a third-line choice. These analgesic classifications are supported by the findings in our study.

We agree that “clinical efficacy is generally poorly predicted by animal models of neuropathy” since their pathophysiology does not reflect what usually happens in patients. We also consider the traditional neuropathic pain assays of mechanical hypersensitivity used for decades in research labs, that we used, to have unrecognized translational merit — as you note they are “rarely used to evaluate analgesic efficacy in clinical trials”, but should be considered in our estimation.

As suggested, an explanation of the validity of the conclusions has been added to the revision in the Discussion.

**References**

1. Finnerup NB, Attal N, Haroutounian S, McNicol E, Baron R, Dworkin RH, Gilron I, Haanpää M, Hansson P, Jensen TS, Kamerman PR. Pharmacotherapy for neuropathic pain in adults: a systematic review and meta-analysis. The Lancet Neurology. 2015 Feb 28;14(2):162-73.
